# Gene co-expression analyses of health(span) across multiple species

**DOI:** 10.1093/nargab/lqac083

**Published:** 2022-11-29

**Authors:** Steffen Möller, Nadine Saul, Elias Projahn, Israel Barrantes, András Gézsi, Michael Walter, Péter Antal, Georg Fuellen

**Affiliations:** Rostock University Medical Center, Institute for Biostatistics and Informatics in Medicine and Ageing Research, Rostock, Germany; Humboldt-University of Berlin, Institute of Biology, Berlin, Germany; Rostock University Medical Center, Institute for Biostatistics and Informatics in Medicine and Ageing Research, Rostock, Germany; Rostock University Medical Center, Institute for Biostatistics and Informatics in Medicine and Ageing Research, Rostock, Germany; Budapest University of Technology and Economics, Department of Measurement and Information Systems, Budapest, Hungary; Rostock University Medical Center, Institute for Clinical Chemistry and Laboratory Medicine, Rostock, Germany; Budapest University of Technology and Economics, Department of Measurement and Information Systems, Budapest, Hungary; Rostock University Medical Center, Institute for Biostatistics and Informatics in Medicine and Ageing Research, Rostock, Germany

## Abstract

Health(span)-related gene clusters/modules were recently identified based on knowledge about the cross-species genetic basis of health, to interpret transcriptomic datasets describing health-related interventions. However, the cross-species comparison of health-related observations reveals a lot of heterogeneity, not least due to widely varying health(span) definitions and study designs, posing a challenge for the exploration of conserved healthspan modules and, specifically, their transfer across species. To improve the identification and exploration of conserved/transferable healthspan modules, here we apply an established workflow based on gene co-expression network analyses employing GEO/ArrayExpress data for human and animal models, and perform a comprehensive meta-study of the resulting modules related to health(span), yielding a small set of literature backed health(span) candidate genes. For each experiment, WGCNA (weighted gene correlation network analysis) was used to infer modules of genes which correlate in their expression with a ‘health phenotype score’ and to determine the most-connected (hub) genes (and their interactions) for each such module. After mapping these hub genes to their human orthologs, 12 health(span) genes were identified in at least two species (ACTN3, ANK1, MRPL18, MYL1, PAXIP1, PPP1CA, SCN3B, SDCBP, SKIV2L, TUBG1, TYROBP, WIPF1), for which enrichment analysis by g:profiler found an association with actin filament-based movement and associated organelles, as well as muscular structures. We conclude that a meta-study of hub genes from co-expression network analyses for the complex phenotype health(span), across multiple species, can yield molecular-mechanistic insights and can direct experimentalists to further investigate the contribution of individual genes and their interactions to health(span).

## INTRODUCTION

Health and healthspan are gaining acceptance as central concepts in medicine, with a focus on (multi-)morbidity, aiming to delay the onset of disease and dysfunction for as long as possible. Health is difficult to describe and has different meanings to different people. Aging, and the deterioration of health that comes with it, affects nearly all species. But tissues that enable the systematic study of the underlying molecular processes are more easily available for animal models, especially for invertebrates, coming with further advantages such as controlled genetics and environments, and a much shorter lifespan. Thus, aging and health are frequently studied in animal models.

To support aging research, many databases are now available (1). Gene expression profiles across tissues of aging mice were already presented, e.g. by the AGEMAP (2) project in 2007 and recently by the Aging Atlas Consortium (3), but there is a lack of such data for health. Adding the dimension of health may amend the identification of molecular markers for aging and further support the identification of health-modulatory compounds (4).

An increasing number of transcriptomic data sets that can be used to compare young and old individuals are available on public repositories. The concept to derive aging-associated patterns from transcriptome repositories across species (5) already led to central elements of aging-related knowledge bases (1,6). Comprehensive analyses of transcriptome repositories were also expanded towards diseases in the context of aging (7). Yet, for expression profiles *per se*, there is a lack of gene expression co-regulation analyses across species with a focus on health(span). A major challenge for polygenic phenotypes in general is the heterogeneity of the underlying gene regulatory landscape (8), impeding the use of network-based methods for post-processing, i.e. smoothing, aggregating, and unifying, transcriptomic results (9,10). However, the power of the cross-species derivation of conserved co-regulation modules is becoming apparent, see, e.g. the CoCoCoNet database (11).

For prominent cellular characteristics of aging, such as cellular senescence, Avelar and coworkers (12) demonstrated how to integrate static data from public databases with insights from gene co-expression (https://coxpresdb.jp/) (13). Attempts have also been made to use known gene/protein interactions to describe age-induced expression profiles (14). The integration of co-expression data, also across species, could similarly be performed with GeneFriends (15) (for human and mouse) for RNA-seq or, for microarray data also with MIM (16). The latter also provides provenance information, i.e. the experimental context in which the correlation was found, to plan follow-up experiments.

We recently proposed an operational definition of health (17) and suggested that it may be applied across species. We then collected data on molecular contributions to health (18), with a focus on genetics. With the support of GeneMania (19) and the associated tool AutoAnnotate (20) we then constructed a map of network modules by clustering a functional interaction network of the genes implicated in health. Naturally, aging and health are complex phenotypes for which we still lack the means to single-out and investigate the contribution of individual genes. A detailed analysis is therefore expected to dissect a list of health-associated genes into gene sets that, in turn, can be understood as parts of the whole (that is, health), and these parts are distributed across diseases & dysfunctions, tissues, organs and species. The idea of identifying health-associated molecular patterns is at the root of molecular health research. Our efforts strived for a consensus across the species barrier between worms (*Caenorhabditis elegans*) and humans, and we investigated the transfer of findings from worms as a short-lived animal model of health to humans. A consensus in network modules of worm and human was thus determined (18), but it was small in relation to the much larger functional interaction networks that were the starting point for each species. However, functional interaction databases, upon which GeneMania is based, are woefully incomplete. Further, these databases do not usually consider the specific biological context of an interaction, but instead merge interaction data from very heterogeneous sets of experiments (8,21).

**Figure 1.**
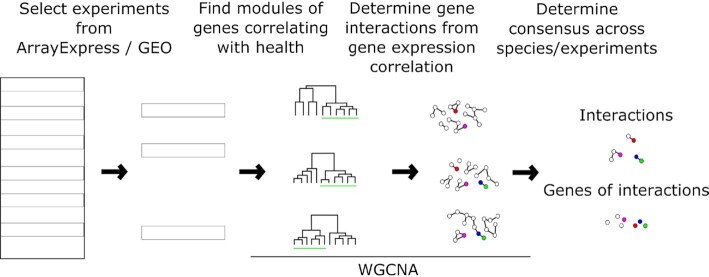
Workflow to determine cross-species consensus gene correlation networks, and subsequent analyses. WGCNA is applied independently for each selected experiment in ArrayExpress/GEO, defining modules and gene interactions. Gene interactions are filtered by experiment-specific thresholds. For each module, hub genes are retrieved and those with an ortholog found as a hub gene in another species are reported in Table 1. For each module, Table 2 lists the genes that correlate the most with its ‘eigengene’, i.e. that best represent the module's expression pattern across samples.

To harness the power of diverse transcriptomic experiments in the context of health(span), here we present a WGCNA-based meta-study for the exploration and characterization of health(span) related modules (Figure 1). WGCNA co-expression analyses have recently been used in aging research (22) to identify differences in old vs young and gene expression asymmetries in the brain that develop over time. In our study we integrated a highly diverse set of health(span) expression data across species from many different tissues. We manually derived a scoring for all the transcriptome samples we considered, based on a score combining quantitative and qualitative factors that the authors of the experiments provided, and refer to it as their ‘health phenotype score’. WGCNA was found to be a competitive tool to find network modules reflecting such kinds of scores (23). This allows the filtering for health-associated modules generated by the WGCNA correlation analysis across tissues (or cell lines) and multiple species, and thus, the meta-study of health-associated most-connected genes (hubs) and their interactions, as presented here. We also collected the evidence for the implication of these genes in health(span) from the literature.

## MATERIALS AND METHODS

### External databases

All sets of transcriptomics experiments in the Gene Expression Omnibus (GEO) (24) and ArrayExpress (25) databases that mention ‘healthspan’ in the title or the description were included, if they featured more than six samples and a scale-free network could be derived from their correlation matrix (for the latter, see below). Experiments performed on *C. elegans* were added when these were alternatively annotated with the term ‘health’, to increase the number of datasets for the worm, since "healthspan and ‘Caenorhabditis elegans’ only finds the single entry E-GEOD-54853. We did not include non-worm experiments with ‘health’ in the title or the description, since the number of matches (specifically for human) turned out to be excessively large.

Log-transformation of expression levels was performed if not already performed for the data we retrieved. Suppl. Table 1 describes the experimental data and metadata which form the input to the following analyses. Our scripts relied directly on expression data provided by the individual uploader. Information on the way the data was normalized is made available in the description of the protocol. A transformation to *Z* scores made no difference to the analysis. We also tried out a quantile normalization (26) for each experiment. Genes still found significant with quantile normalization applied are indicated as such in Tables 1 and 2.

**Table 1.** Hub genes in health-associated WGCNA network modules, found in at least two species. Orthologs were mapped to the human gene name using Ensembl. The human gene names also correspond to the names in mouse and rat, whereas the names of the orthologs in worms based on the Ensembl database are given in brackets. The last column summarizes the report by the OGEE database labeling the gene as essential. If available, it presents what fraction of cell-culture experiments showed the gene to be essential across the many human tissues of the avana set of experiments. PPP1CA and SDCBP (marked by an asterisk) have also been found as hub genes when starting with quantile-normalized data, see Supplement Table S3, which also lists other members of the MRPL and SCN families

Gene human (worm)	Human	Mouse	Rat	Worm	Description in context of healthspan	Essential (OGEE)
ACTN3 (atn-1)			x	x	Expressed in muscle, known marker for healthspan and athletes’ muscle phenotypes (36). Localized to Z-discs, anchoring to actin filaments.	no
ANK1 (unc-44)		x		x	Ankyrin 1 (ANK1) is associated genetically with Diabetes type 2 (37), spherocytosis (38) and epigenetically with neurological diseases, likely triggered by ApoE with effect on TNFalpha and Akt (39).	conditional (mouse)
MRPL18 (mrpl-18)		x		x	The mitochondrial ribosomal protein L18 (MRPL18) is involved in the cytosolic stress response and promotes the translation of Hsp70 (40).	10–27%
MYL1 (mlc-6 & mlc-5)	x		x		MYL1 encodes the myosin light chain 1 expressed in fast-twitch skeletal muscle fibers (41). Human ageing is associated with lower MYL1 content and higher MYL3 content (42).	4–38% (broad)
PAXIP1 (pis-1)		x		x	The PAX interacting protein 1 (PAXIP1) contributes to DNA repair and correlates with breast cancer staging (43).	2–18%
PPP1CA* (C06A1.3 & 26 others)	x			x	PPP1CA is one of three catalytic subunits of the serine/threonine specific protein phosphatase 1 (PP1), which is known to be involved in the regulation of glycogen metabolism, cell division, muscle contractility and protein synthesis (44). PPP1CA itself is linked to diverse tumor entities and is involved in ERK/MAPK signaling (45, 46), TGFβ signaling (47), Ras signaling and Ras-induced senescence (48), spermatogenesis (49) as well as in tau hyperphosphorylation leading to Alzheimer's disease (50).	1–21%
SCN3B (-)		x	x		The sodium voltage-gated channel beta subunit 3 (SCN3B) controls electrolytes and contributes to the pacemaking in the heart and has an effect on intracellular trafficking (51). It also suppresses senescence and apoptosis via its interaction with p53 and thus, is considered to be an oncogenic factor (52).	no
SDCBP* (lin-10)		x		x	Syntenin-1 (formerly Syndecan(SDC)-binding protein) regulates autophagy (53) and together with Syndecan contributes to exosome formation (54) also in cancer cells (55).	3–21%
SKIV2L (skih-2)	x		x		The Ski2-like RNA helicase (SKIV2L) is part of the Super killer (SKI) complex and involved in mRNA degradation, DNA-RNA hybrid control, and telomere stability (56). SKIV2L is also known to contribute to inflammatory bowel disease (57) and macular degeneration (58). Furthermore, SKIV2L features antiviral capacities and plays a role in innate immunity (59) associated with RNA exosomes (60).	no
TUBG1 (-)	x		x		TUBG1 encodes the tubulin gamma 1 protein, which, when mutated, can lead to brain malformations (61) with clinical features such as motor and intellectual disabilities and epilepsy. Moreover, TUBG1 is involved in tumor diseases, as shown for breast cancer (62), lung cancer (63) and medulloblastomas (64).	common 60–100%
TYROBP (-)		x	x		The transmembrane immune signaling adaptor TYROBP is considered to be involved in Alzheimer's disease (65,66) and as a target of TERC in inflammatory processes (67). In addition, TYROBP is suggested as a prognostic marker for gastric cancer and renal cell carcinoma (68, 69).	9–20%
WIPF1 (wip-1)		x		x	The WAS/WASL interacting protein family member 1 (WIPF1) regulates actin, phagocytosis, and neurotransmission and is among the top-3 genes upregulated by caloric restriction in the hypothalamus of wild-type mice (70). Furthermore, overexpression of WIPF1, triggered by BRAF-mutation activated MAP kinase pathway, promotes aggressiveness of thyroid cancer and thus acts like an oncoprotein (71). Its oncoprotein character was also described for pancreatic adenocarcinoma (72) as well as breast cancer, glioma and colorectal cancer (73).	no

**Table 2.** Genes correlating the strongest with the module's eigengene (quantifying module membership) in at least two species. Genes in this table are among the top-30 of the module membership and found in experiments of at least two species. The gene name is marked in bold if that gene was listed as a hub gene in Table 1. The column ‘Correlation’ flags ‘positive’ (or ‘negative’) to refer to an observed positive (or negative) correlation with the ‘health phenotype score’ when the gene is upregulated. ‘mixed’ indicates that the experiments did not yield a consensus direction of correlation. Supplement Table 2 extends this list to all genes that appear in the top 30 of modules of two or more experiments. The ‘#Experiments’ column indicates the number of experiments with a module for which the gene was identified as a member. The last column summarizes the report by the OGEE database labeling the gene as essential as in Table 1. *PPP1CA and SQSTM1 (marked by an asterisk) have also been found as hub genes when starting with quantile-normalized data, see Supplement Table S3, which also lists other members of the MRPL and SCN families. ^+^CEBPB, PAXIP1 and PPP1CA (marked by a plus sign) are confirmed by the same analysis on quantile-normalized data presented in Supplement Table S4

			Correlation with healthspan phenotype					
Gene		#Experiments	All species	Human	Mouse	Rat	Worm	Essential (OGEE)
AC068831.7	vps-33.2	2	*negative*		*negative*		*negative*	conditional (worm)
ADAM10	sup-17	2	*mixed*		*negative*		*positive*	no
APBB1IP	mig-10	2	*mixed*		*negative*		*positive*	no
CEBPB^+^	cebp-1	2	*mixed*		*negative*	*positive*		no
CREBBP	cbp-1	3	*negative*		*negative*		*negative*	2–11%
EIF3F	eif-3.F	2	*positive*		*positive*		*positive*	yes (sanger)
INTS12	F53H1.4	2	*mixed*		*negative*		*positive*	1–21%
KPNA3	ima-3	2	*mixed*		*negative*		*positive*	11–25%
MEX3C	mex-3	2	*negative*	*negative*			*negative*	no
MRPL19	mrpl-19	2	*mixed*		*positive*		*negative*	2–26% (broad)
**MYL1**	mlc-6	2	*positive*	*positive*		*positive*		4–38% (broad)
**PAXIP1^+^**	pis-1	2	*positive*		*positive*		*positive*	1–21%
PCNX2	B0511.12	2	*negative*		*negative*		*negative*	conditional (mouse)
PPP1CA*^+^	C06A1.3	4	*mixed*	*positive*			*mixed*	8–20%
PPP1CB	gsp-1	2	*positive*			*positive*	*positive*	52–96% (sanger)
PPP2R3C	-	2	*negative*	*negative*	*negative*			10–27%
RAB2A	unc-108	2	*negative*		*negative*		*negative*	no
RAB31	-	2	*negative*		*negative*	*negative*		no
RPL29	rpl-29	2	*positive*		*positive*	*positive*		2–30% (broad)
RTN2	-	2	*mixed*	*positive*	*negative*			no
RYR1	unc-68	2	*positive*	*positive*	*positive*			no
**SCN3B**	-	2	*positive*		*positive*	*positive*		no
SIX4	ceh-32	2	*negative*		*negative*		*negative*	no
SNRPD1	snr-3	2	*negative*		*negative*		*negative*	common 100%
TMEM70	F32D8.5	2	*mixed*		*positive*		*negative*	no
**TUBG1**	-	2	*mixed*	*positive*	*negative*			common 60–100%
**WIPF1**	wip-1	3	*negative*		*negative*		*negative*	no
ZC3H15	F27D4.4	2	*negative*		*negative*		*negative*	no
SQSTM1*	sqst-1	3	*negative*		*negative*	*negative*	*negative*	2–25% (broad)

Data on gene essentiality was retrieved from the Online GEne Essentiality (OGEE) database (27). OGEE describes for several large initiatives what fraction of cell-culture experiments per tissue were inhibited in growth if a gene was disabled. If available, the minimal and maximal fractions are presented for the many human tissues of the ‘avana’ set of experiments, ‘broad’ or ‘sanger’ were alternatives. There was no gene for which OGEE did not have data provided by one of the sources.

Each experiment's metadata was inspected to manually derive a health(span) phenotype score designed to reflect the health status of the individual(s) from which the sample(s) were taken. This ‘health phenotype score’ was manually tailored for each experiment by a custom formula that takes the experiment's factor annotation as an input and thus consistently annotates each sample: For our analysis, if an experiment provides phenotype scores that reflect the health of the individual samples, then these scores are taken. If such a score is not available for an experiment, it is derived by a minimal number of operations from the phenotypes or from the experimental factors describing the experiment. Such a minimal operation could be the inversion of a factor describing accumulated damage. If multiple factors are given (like a combination of treatments), then we inspected the paper in detail to derive a combined score. Gene knock-downs, knock-outs, gene transfers or natural genetic variations are all interpreted as if they were a treatment. The young untreated wildtype individual was assigned a health phenotype score of 1. If a treatment was reported in the paper to improve or reduce health, by default we added or subtracted 0.2, for example yielding a score of 1.2 for the healthier individual. Importantly, only the scores of the individuals providing the samples within one experiment must be comparable to each other and we never relate the scores in one experiment to the scores in another experiment. This allows us to use a rather arbitrary scorecard that has to be consistent only within one experiment. Within such an experiment, scores may be adjusted further considering the relative strengths of the effects reported in the paper. Samples from old individuals are assigned a healthspan phenotype score of 0, which may be increased or decreased by 0.2 as before. The details can be inspected in the ‘Data_parameters’ folder (see Availability).

### RNA-seq data re-analysis

Gene expression levels were typically not available for the RNA-seq data. Therefore, the RNA-seq datasets were all reanalyzed based on the raw data by the following protocol. All target RNA-seq datasets were retrieved from the European Nucleotide Archive (28), and the corresponding FASTQ files were filtered for Illumina adapters, phage PhiX sequences and quality (Phred score over 25) using BBTools version 38.49 (29). Gene expression was then quantified for each RNA-seq run. To this end, the filtered outputs were mapped against the corresponding target genomes from the Ensembl database release 98 (30), using the STAR program version 2.7.3a (31). This program also enabled us to assign uniquely mapped reads to individual genes from the short read alignments. Finally, the mapped read counts were normalized as transcripts per million (32).

### Network analysis

In WGCNA, the network construction is independent from the separation of genes into modules: The modules are a means to perform a gene selection and prioritization with respect to an observed association with the phenotype. The interaction network is defined via a threshold on a score on pairs of genes, which is not the Pearson correlation itself. Instead, it is a score that indicates to what degree a pair of genes correlates in the same fashion (in terms of direction and magnitude) with other genes (33,34). The scored gene pairs are filtered for a minimum interaction score of 0.2.

The WGCNA analysis was performed for undirected interactions. Parameters were set as instructed by the WGCNA standard protocol, as follows. For every experiment the *cutHeight* was manually set to remove outliers and the exponent/power was manually determined to ensure that the network is a scale-free network (see below). An experiment is skipped if that is not possible and then marked with ‘no modules found’ in Supplementary Table S1. For RNA-seq, prior to the removal of outliers, low-count genes were removed by a manual setting of the parameter *cutHeight* so that the separation of the samples reflects their phenotypes and could no longer be improved, based on the clustering of the genes by expression data with the R function *hclust* as performed as part of the WGCNA protocol.

The WGCNA protocol proposes to apply an experiment-specific exponent to the correlation coefficients (WGCNA calls it ‘power’) to strengthen the differences in the correlation data. This power is set, for each experiment, just large enough so that in the derived correlation network, the fraction of genes that have *k*-many interactions with other genes is proportional to k^−γ^ with **γ** being a small positive parameter. Networks with that property are called scale-free; the parameter **γ** describes how quickly this fraction gets smaller when the number of connections increases. Genes with a high number of connections are rare in scale-free networks, but they exist, and these ‘hub’ genes are considered highly influential on the expression levels of genes in that module. Further, we filtered for modules that are associated with the health(span) phenotype (see next paragraph), and the hub genes are likely to also have a strong effect on this phenotype.

Only network modules whose WGCNA eigengene correlated with the ‘health phenotype score’ (*P* value < 0.05) were retained further. Then, the 30 genes (see the WGCNA tutorial https://horvath.genetics.ucla.edu/html/CoexpressionNetwork/Rpackages/WGCNA/Tutorials/) most connected in a module according to the WGCNA *softConnectivity* function were considered for subsequent consensus analyses, and called ‘hub’ genes hereafter. Besides the modules, output of the WGCNA workflow is the topological overlap matrix with a quantitative description (termed *adjacency*) of all interactions between any pair of genes of an experiment. For each experiment, we determined a threshold at the 95% quantile of all the adjacency values. Only gene interactions with an adjacency above that experiment-wide threshold contribute to our analysis of interactions of genes in the health-associated modules. For the 30 hub genes, all pairwise interactions above that experiment-wide 95% quantile were thus exported, i.e. subjected to a pan-module search for consensus genes and consensus interactions, also across species by considering orthologs, presented in Tables 1 and 2. Orthologs were determined based on Ensembl version 101 (30).

Further, for each health-associated module, the 30 genes correlating the strongest with the module's eigengene (reflecting average module behavior, also called ‘module membership’ in WGCNA) were retrieved. Those found in at least two species are presented in Table S3. The correlation is taken in relation to the eigengene, and not in relation to the health phenotype score. Either would be fine for a ranking of the hub genes within a module, and the ranking is expected to be identical for the genes most central to a module. However, our particular interest was to abstract from the phenotypes of the experiment and thus utilize the WGCNA-performed modularization to influence the ranking. This is assumed to be particularly useful for experiments with multiple health-associated modules, each of which we expect to focus on a different aspect of health and for which the constituent genes should thus be ranked differently, in order to analyze that particular module most appropriately, and without any particularities pertaining to the health phenotype score. Multiple probesets describing the same gene, or its splice variants, were not distinguished and mapped to the same human gene, resulting in genes interacting with themselves. Such self-interactions were removed.

### Network figures

**Figure 2.**
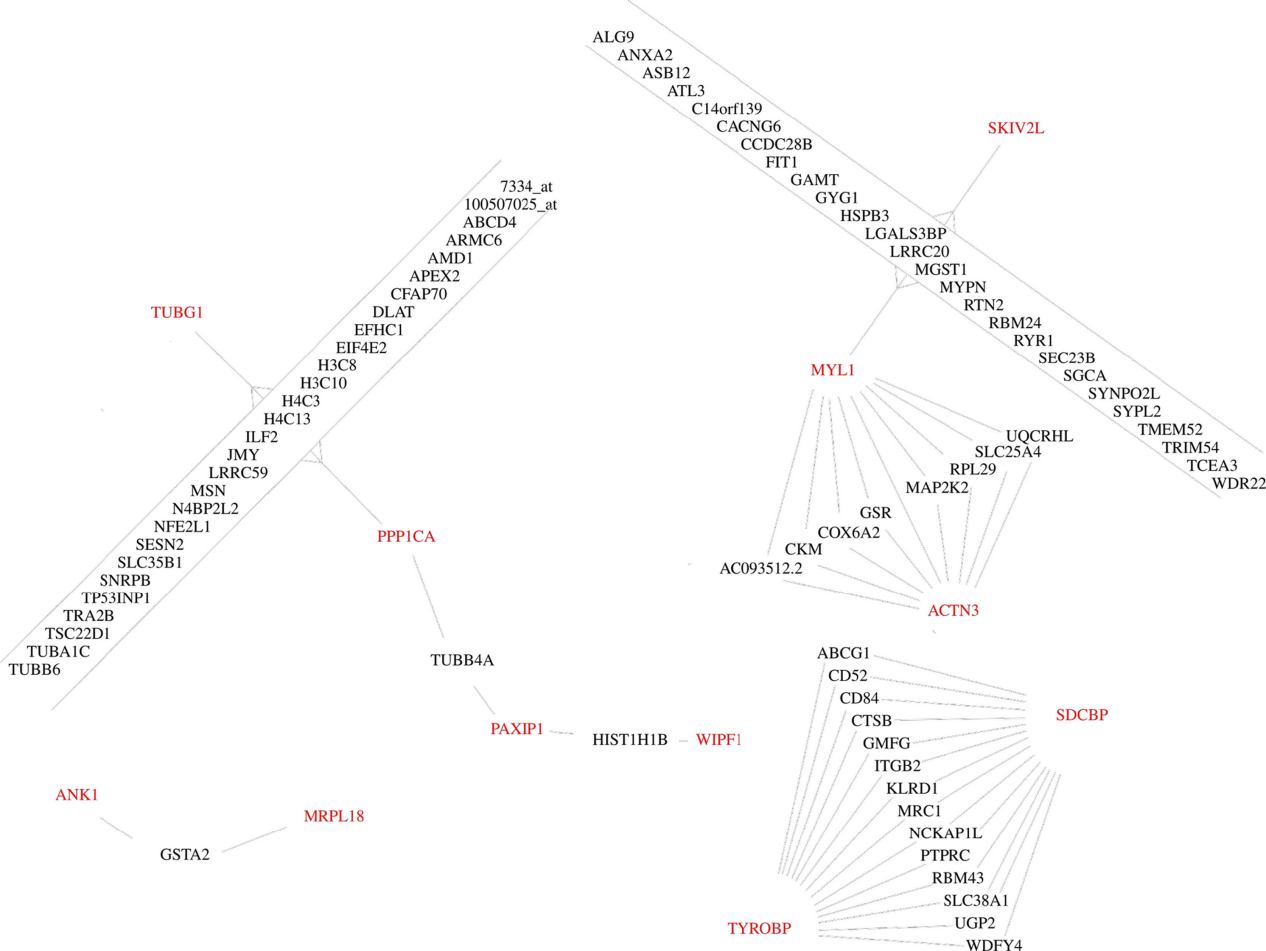
Cross-species conserved hub genes observed in health(span)-associated WGCNA modules, and genes that connect these hub genes. Connections are interactions taken from the WGCNA adjacency matrix if the adjacency is above the 95th percentile of all interactions of that experiment and if for that experiment the interaction is in a health(span)-associated module. The only direct interaction between hub genes is between MYL1 and ACTN3.

The network figures were created with the R igraph package. A spring-embedding layout was chosen for the plots and manually refined. Figure 2 shows an overview on all hub genes from Table 1, their direct interactions and genes found in any species that connect to at least two hub genes. Additionally, Figure 2a–d in the Supplement were prepared separately for each species, i.e. they show only interactions from modules that WGCNA identified for an experiment based on samples from that species. Input to these supplement figures are the hub genes from Table 1 and all genes that are reachable from the hub genes which are no more than two transitions away. All interactions between the selected (reachable) genes were also added. The resulting graphs were simplified with the igraph minimum spanning tree implementation that maintains the connectivity of the graph but removes all redundant paths between genes. The spanning tree retains the stronger of two alternative paths between genes. A gene connected to a hub gene with a low adjacency value will thus lose that direct link if it is correlating strongly with another gene that has a strong correlation with that hub gene. Hub genes were determined from within WGCNA considering all interactions, not only the ones above the 95th percentile. Hub genes that are strongly connected for experiments in one species may not be equally dominating in another species. This possibility and the competitive effect on directly connected hub genes (one cross-species, the other only observed for one species) imposed by the spanning tree give the impression that the cross-species consensus hub genes are marginalized in Supplemental Figure S2a–d, albeit these graphs are seeded from the consensus hub genes and their interactions.

### Permutation tests

The question whether the selected hub genes (see Table 1) are special with respect to their number of interactions in protein-protein interaction databases was addressed empirically. Hereto, all genes in the STRING (35) database were ordered by their degree (sum of incoming and outgoing connections). For single genes a rank in that order can be determined, and for any set of genes the sum of these ranks can be derived. 10 000 random gene selections of equal size each yield their respective rank sum. The fraction of these random gene selections with a higher rank sum score than the gene sets in Tables 1 and 2 estimates the probability to make that respective finding by chance.

Similarly, the significance of the number of consensus genes of Tables 1 and 2 was assessed by repeating the consensus determination with an equal number of randomly picked genes from the respective same species.

## RESULTS

We analyzed all experiments listed in Supplemental Table S1 with WGCNA. This analysis provided a modularization by an expression-based clustering of genes and allowed to describe the association of each module with the ‘health phenotype score’. WGCNA also quantified the strength of gene correlations and determined hub genes for each module. We identified 12 genes (Table 1) that are among the 30 hub genes in health(span)-associated modules from at least two species. In total (Supplement Table 2), 658 different genes were found among these top-30 hub genes of all modules as determined by WGCNA. An interaction network of the genes from Table 1, based on correlation of gene expression, is presented in Figure 2.

To prioritize the cross-species hub genes of Table 1, we also looked at the module membership of all genes for each module. The genes most correlating with the module's eigengene are reported and, analogous to Table 1, the genes that are found in multiple species were determined and listed in Table 2. This table further indicates whether a gene's change in expression is positively or negatively correlated with the eigengene of the WGCNA module to which it belongs, which in turn may be positively or negatively correlated with the health(span) phenotype. Supplement Table 2 shows the WGCNA module details from which Table 2 was derived. To allow for a direct comparison of the genes’ correlation with health(span), not quantitatively but in terms of direction (that is, up- or downregulation in relation to the health phenotype score), Table 2 presents a gene's inverted direction if the gene's module is already negatively correlated with the health(span) phenotype. Its column ‘ Correlation’ presents the direction that all experiments are in agreement with or ‘mixed’ if the experiments differ in terms of their correlation with the health phenotype score. This information can be calculated for all genes, which we consider to help interpreting a module. The provenance of each module is described in the supplement (Supplement Table S1).

**Figure 3.**
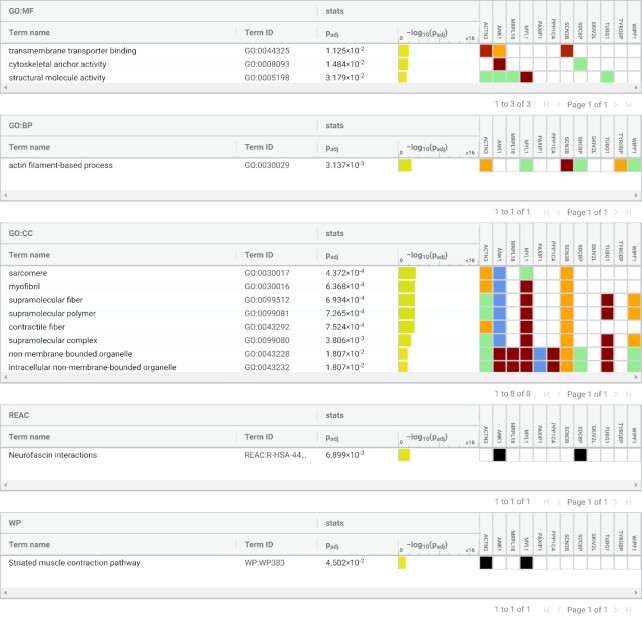
Gene set enrichment analysis of cross-species hub genes for health(span) with g:profiler. Input are genes from Table 1 that are observed in healthspan-associated modules of multiple species. Terms with a low coverage of genes are not suitable to describe the selection as a whole but may still direct the interpretation of parts of the network where these genes are connected. Color codes are used for the *P* value (4th column) and color represents the source for the gene assignment (5th column), cf. the g:Profiler documentation, https://biit.cs.ut.ee/gprofiler/page/docs.

The intersection of Tables 1 (hub genes) and 2 (genes correlating with the health phenotype score) points to a subset of genes that are considered both influential and directly associated with health, i.e. MYL1, PAXIP1, PPP1CA, SCN3B, TUBG1 and WIPF1. The enrichment by g:profiler for the genes of Table 1 are shown in Figure 3. Supplement Figure S1a shows an enrichment analysis for the intersection of Tables 1 and 2 which is matching closely the enrichments in Figure 3, except that it does not feature the terms associated with muscle. Supplement Figure S1b shows the enrichment for all genes in Table 2. The latter is the least robust since the enriched terms do not cover a large fraction of the genes.

Tables 1 and 2 were both created by taking the 30 top-ranked genes in each module. The gene set enrichment analyses for Table 2 (Supplement Figure S1b) were not conclusive but the 29 genes (Table 2) in multiple species are unlikely to be achieved by chance, as we demonstrate next. The quantiles after 10000 permutations with an equal number of random genes selected for a module are

**Table:** 

0%	1%	5%	10%	25%	50%	75%	90%	95%	97.5%	99%	99.5%	99.9%	100%
0	3	4	5	7	9	11	13	14	15	16	17	19	21

which indicates a *P* value < 0.0001 to find 29 genes confirmed as healthspan-associated across species with modules of the same size and a maximal selection of 30 genes per module. Analogously, for the selection of hub genes, an equal selection of random genes after 10 000 iterations shows this distribution:

**Table:** 

0%	1%	5%	10%	25%	50%	75%	90%	95%	97.5%	99%	99.5%	99.9%	100%
0	1	2	3	4	5	7	8	9	10	11	12	13	16

This indicates a *P* value < 0.01 to confirm 12 hub genes across species by chance from modules of equal size.

We also tested if the genes in Tables 1 and 2 have more interactions than one could expect by random selections of an equal number of genes in STRING. For interactions with a quality score of 900, which excludes genes that have only support by gene-correlation analyses, a *P* value of 0.0022 was empirically determined with 10000 repeated attempts to outperform the hub gene selection of Table 1, while the *P* value was found to be 0.019 (not significant) for the genes of Table 2. When lowering the quality score threshold to 200, as suggested in the tutorial of the R interface of the STRING database, then both gene selections are found to be significant with *P* < 0.0001.

### Effect of data normalization

WGCNA is considered robust towards linear transformations that affect all genes in an equal fashion since only the correlation between genes affects its data processing. External factors that increase or lower the expression of all genes in a dataset will only increase the threshold above which a correlation becomes significant. Thus, a shift of the mean, also combined with a division to derive *Z* scores does not affect the analysis.

We also performed quantile normalization which, for each gene, examines the data across samples and which therefore does not necessarily maintain the order of expression levels for each sample. This affected the correlation scores, the modules and the module membership ranking for each module, and the results. Data for the same analysis performed on quantile normalized expression levels are shown in the supplement. For our analysis we used the data as normalized by the authors of the respective dataset. This should enable the straightforward comparison of the analyses with the publication accompanying the data and it leaves the decision regarding the normalization method and correction for batch effects to the authors of the dataset, who are much more familiar with it.

## DISCUSSION

### Method

The basis of this investigation were all experiments in GEO/ArrayExpress that mention ‘healthspan’ in their description (or ‘health’ or ‘healthspan’ in case of worm). For each experiment, from the descriptions that are provided for the samples in the database, a ‘health phenotype score’ was derived. A gene expression correlation analysis with WGCNA yielded a gene coexpression network for each experiment as a set of modules of genes that correlate with the health(span) phenotype. We were interested in genes that are most connected, i.e. hub genes, for each module, and in their interactions as described by the WGCNA network. The correlation of genes with the module eigengene (Table 2), to predict a positive or negative association with health in the molecular context of that module, was only of secondary interest to us.

In this analysis, we focussed on common observations across two or more species and a variety of health-related phenotypes, including the reaction to drugs that extend healthspan (Supplementary Table S1). The first steps of our analysis with WGCNA identified modules directly from the expression data, i.e. without inspecting a phenotype; the selection of health(span)-associated modules was performed in a later step. The WGCNA protocol was directly derived from the WGCNA tutorial.

The selection of genes, based on strong connectivity, from modules selected in such a way shall hence be considered robust even if the mapping of the multi-factorial sample descriptions to a single factor, that is, the health phenotype score describing the health-effect observed in samples, may allow for plausible alternatives. This is another reason, besides the need for abstraction to compare experiments, why we consider it advantageous to compare the module's genes against the module's eigengene, which is derived solely by an inspection of the expression data, and not against the health phenotype score (as done in Table 2). The manual intervention to derive the health phenotype score was solely needed to filter for health(span) associated modules (Supplementary Table S2).

To filter for gene interactions, we decided to filter for the strongest 5% of adjacencies from each experiment, further constrained to modules that are associated with the health(span) phenotype score; see the Methods section for details. This experiment-dependent threshold reflects that experiments differ in the number of samples and subgroups and hence in the contrasts to separate genes by their correlations.

The authors of WGCNA suggested that their software can be used to perform network meta-studies from multiple microarray experiments in a single WGCNA setup (74). But they clearly stated that the same module needs to be robust across experiments to directly perform WGCNA on a single joint matrix based on all expression data. For the very diverse set of experiments contributing to our analysis and their polygenic phenotype this is not necessarily expected to be the case, i.e. experiments may have their true healthspan-associated module in different sections of the transcriptome. Indeed, we did not observe any interactions to have orthologs across species. The setup presented here is pragmatic and robust, i.e. individual experiments can be removed without affecting the gene interactions determined for another experiment. Of major concern for us was that hub genes are expected to show a measurable effect on health(span) only under the conditions of those ArrayExpress/GEO experiments in which they are differentially expressed. To follow this work up with wet lab confirmations, it is hence essential to provide provenance information on how the change to the hub gene's expression was induced, i.e. a pointer to the ArrayExpress/GEO experiment. In a joint matrix across many experiments this information would be more difficult to retrieve, which suggests not to conduct the integration of experiments directly within a single WGCNA analysis.

Furthermore, for integrating interaction data from multiple experiments, the authors of WGCNA suggested to weigh the interactions from each experiment to derive a single joint adjacency matrix and they suggested to apply a threshold on that single matrix to derive a network. Because of the heterogeneity of our experiments, we cannot tell which experiment would be more informative for health(span), compared to another, and thus could not adjust weights accordingly. By treating all experiments individually, with the null hypothesis that all experiments have the same fraction of true interactions that shall be identified by the respective highest adjacency values, we could use an experiment-tailored threshold for filtering the interactions. Therefore, we used the 95th percentile of correlation values in the adjacency matrix, for each experiment, to adapt the selection of the interactions to be forwarded to describe a meta-study consensus (see Figures 1 and 2). These gene interactions may be trusted and they thus could be reassembled into a larger integrated meta-study network to reflect the molecular neighborhoods of hub genes, which we presented as Figure 2 (cross-species) and Supplement Figures 2a-d (for multiple modules of the respective same species). The comparison of findings across species further strengthens the confidence in the WGCNA results. Thus, we identified conserved candidate regulators of health(span).

An important technical concern lies with the interpretation of gene expression correlation data for RNA-seq experiments, which have an intrinsic high noise-level for low-abundant genes. We have shown (75) that even for array data (that are less noisy for low-abundant genes), the low-abundant genes have a measurable effect on a ranking of genes by Pearson correlation, and this is likely also the case for module calculations as performed here. This concern has to be borne in mind in the following interpretation of the modules in terms of biological functionality.

### Cross-species hub genes and their interactions

Most of the hub genes identified by our analysis (Table 1) have been described in a health(span)-context before. The gene set enrichment analysis with g:profiler describes the molecular roles of the cross-species hub genes (Table 1) as specifically associated with a) features of the muscle and b) actin filament-based organelles and movement (Figure 3). The worm is a model species also for muscle development because of striking similarities of its muscles to mammalian muscle tissue (76), and movement (locomotion) is an important phenotype in all species towards operationalizing health by quantification (17). For human, rat and mouse in Supplementary Table S1, there are experiments for which samples were selectively taken from muscle tissue, but not so for the worm, which is routinely sequenced as a whole. Upon closer inspection of the enrichment results of Figure 3, we found that ‘actin filament-based movement’ refers to a wide spectrum of processes, i.e. genes that support actin polymerisation (WIPF1), the motor protein myosin (MYL1) or the transition of endosomes into exosomes for intercellular communication (SDCBP).

The number of experiments of vertebrates and invertebrates is balanced. Apart from a lack of tissue specificity, the experiments for the worm differ from rodents and humans, in that worm experiments may comprise samples from different larval stages. This may ease the task of finding strong correlations between genes, but specificity for aging-associated processes is likely reduced.

Inspecting the distribution of hub genes by species, we found no more than five of the 12 hub genes in worm, cf. Supplement Figure S2b, and four in human, cf. Supplement Figure S2a. The only conserved direct interaction between consensus hub genes was observed between MYL1 and ACTN3 (Figure 2). However, interactions were found multiple times for experiments of the same species, namely ABRA with VRK2, AQP11 with GSTA2 and CYLD with PCNX2 for the worm. These three interactions are shown in Supplement Figure S2b and the VRK2 gene remains directly connected with the PPP1CA hub gene also after the minimum-spanning-tree-based edge removal. VRK2 is described to have downstream effects on the consensus hub gene PPP1CA (77) via GSK3beta (78). Its genetic variants are associated with a series of neurological diseases and viral infection, but also with healthspan associated sleep patterns (79). The interactions conserved in multiple species are not confirmed in STRING for humans, however in worms, the consensus hub gene PPP1CA (C06A1.3) links to VRK2 (tag-191).

By interpreting the enrichments in Supplement Figure S3 we can gain more insight into how the genes we identified may be involved in health. An example is the enrichment referring to the TYROBP pathway described in wikipathways and to the GO term Leukocyte activation (Supplement Figure S3a). Genes connecting MYL1 and SKIV2L are involved in muscular structures (Supplement Figure S3b). Tubulins (e.g. TUBG1) are known to bind to PP1, of which PPP1CA is a subunit and together these proteins regulate histone acetylation (80), which is reflected by the genes connecting PPP1CA and TUBG1 (Supplement Figure S3c). Further, enhanced histone acetylation is associated with extended health and lifespan in worm (81).

The highly connected genes selected in this study differ from the list we recently published (18). This WGCNA-based study does not refer to prior knowledge about genetic contributions and does not perform a factor analysis to directly associate genes with a health(span) phenotype. Instead, our focus here is the network-centric interpretation of correlations within gene co-expression clusters, i.e. WCGNA modules. It is the module as a whole that correlates in its expression with health, not necessarily the individual genes. Table 2 lists genes within the clusters that are most representative for the features/characteristics of the cluster in question, i.e. that have the highest degree of *module membership* by WGCNA definition, and in the table, there are marks (by boldface) for the subset of genes that are also hub genes. Of the cross-species hub genes in Table 1, six are also listed in Table 2. Others are ‘near misses’, e.g. Table 2 does not list the consensus hub gene MRPL18 but MRPL19. And besides the consensus hub gene PPP1CA, other PP1 subunits like PPP1CB and PPP2R3C are found in two species (Table 2). The PP1 subunit PPP1R8 was found as a hub gene only for the worm (Supplement Figure S2b).

In Table 2, we report SQSTM1 as the only gene that is associated with health in three species. That gene was long suggested to be aging- and health-related (82,83), also for human, even though it was only found to be health(span) associated in the animal experiments of this study. Its transcript is negatively correlated with health, but SQSTM1 overexpression is known to extend healthspan in worm (84), which may be suggestive for a protective upregulation effect.

We performed the permutation test as a means for an internal validation of the analysis. The re-analysis on a differently normalized dataset means that any consensus is given with extra confidence in the association of PPP1CA with healthspan (this association was found in all runs with only 30 genes exported, for interactions and as a hub gene). Extending the quantile normalization's export to 60 genes also confirmed CEBPB, PAXIP1, and SQSTM1 with healthspan. If exporting 60 instead of 30 genes per module for the original data (data not shown) and for quantile normalization (Supplement Tables S3 and S4), the consensus grows to ABCD4, ANXA2, BAP1, GDF15, PPP1CA, SDCBP and SQSTM1, for which g:profiler finds an enrichment for ‘exosome formation’.

In a review by Bartha and coworkers (85) a gene is defined as essential when its dysfunction has a strong effect on an individual's viability/fitness. Healthspan is also influenced by resilience, which without respective environmental stimuli will not show its effect on fitness and hence is less likely to be identified by knock-out/down experiments in vitro. Nevertheless, we provide references to the OGEE database for all genes in Tables 1 and 2. Most genes are found essential in a fraction of cell cultures, both for the selected hub genes and the genes that highly correlate with the healthspan phenotype. The OGEE database will keep growing, but if no indication of essentiality has yet been reported and a gene is positively correlated with the health score then this may be an early indication for a gene to be simply repairing or otherwise strengthening an individual's resilience. We also cross-checked with the GTEx database for genes that appear equally present across tissues and gender, which would be the expected characteristics for housekeeping genes. To represent the common notion of housekeeping genes, we combined multiple scores including the proportion of samples that express a certain gene as well as the gene's mean expression and its standard deviation (86). This finds the genes from both Table 1 and Table 2 to be enriched (Wilcoxon rank sum test *P*_Table 1_ = 0.0001 and *P*_Table 2_ < 0.0001) for their ubiquity across tissues. The most ubiquitous genes of Table 1 are PPP1CA, SDCBP, and MRPL1, of Table 2 these are RPL29, PPP1CA and PPP1CB.

We could not increase the number of health-associated modules (or genes within modules) by lowering the minimal module size that was defaulting to 30. Nevertheless, the low *P* values from the permutation tests give extra confidence in our findings. It should however be noted that the WGCNA tutorial's limit on 30 genes and the threshold imposed on the interaction score were key to achieve these significance levels. With more genes exported per module this increases the likelihood to find a match in another species by chance. With the quantile normalization applied we could double the export per module to 60 genes while remaining significant in the permutation test with 67 hub genes in the consensus across species (*P* < 0.001, 50% quantile at 44, Supplement Table S3).

Overall, our meta-analysis of a highly diverse set of transcriptomics experiments successfully identified genes which, for the most part, were already established to be closely associated with health(span), and together they have a strong and meaningful GO term enrichment. The enrichment of muscle-related genes can be credited to our focus on health(span) experiments, and our study found many ‘actin filament-based movement’ genes (Figure 3) that provide the cellular infrastructure not just for movement, but also for signaling and cell division, which may be triggered/blocked whenever cells start to feel unwell. If so, then it may be possible to detect many healthspan genes solely by inspecting cellular data. This hypothesis may be confirmed by an extension of our setup to a larger set of cellular transcriptomics data sets for which samples vary in their genetic or environmental exposure to stress factors.

This study provided a cross-species meta-study of gene interactions for health(span)-related datasets in ArrayExpress/GEO. It focused on a series of co-expression network analyses and subsequently on derived hub genes, instead of a focus on those genes that correlate the most with the ‘health span phenotype score’. Tissues, technologies and experimental setups differ between the experiments but are homogeneous for each WGCNA analysis performed. This approach allows for an abstraction from the experiment at hand and permits a search for common mediators of an effect. The proposed consensus hub genes were plausible in their implication into health(span). Their interactions could be confirmed in STRING, or were found consistent with gene set enrichment analyses and they may support the interpretation of joint or epistatic effects between pairs of haplotypes in healthspan GWAS or linkage analyses. The protocol as provided with WGCNA is very transparent so that findings can be traced back to the experiments that are backing them, to serve as a template for further investigations in the wet lab.

## DATA AVAILABILITY

Our implementation is available online at https://bitbucket.org/ibima/healthspantranscriptomicsnetworks/.

## Supplementary Material

lqac083_Supplemental_File
