# Supplementary material for: Gene co-expression analyses of health(span) across multiple species
Source: NAR Genom Bioinform. 2022 Nov 29;4(4):lqac083. doi: 10.1093/nargab/lqac083 (PMC9706456; doi:10.1093/nargab/lqac083)
Supplement: lqac083_Supplemental_File [file lqac083_supplemental_file.pdf]

Gene co-expression analyses of health(span) across multiple species

Steffen Möller[1*], Nadine Saul[2], Elias Projahn[1], Israel Barrantes[1], András Gézsi[3], Michael Walter[4], Péter Antal[3], Georg Fuellen[1]
Earlier version submitted to https://doi.org/10.1101/2021.04.08.439030

[1] Rostock University Medical Center, Institute for Biostatistics and Informatics in Medicine and Ageing Research, Rostock, Germany
[2] Humboldt-University of Berlin, Institute of Biology, Berlin, Germany
[3] Budapest University of Technology and Economics, Department of Measurement and Information Systems, Budapest, Hungary
[4] Rostock University Medical Center, Institute for Clinical Chemistry and Laboratory Medicine, Rostock, Germany

*corresponding author

Supplement

Figures

GO:BP Term name	Term ID	p_{adj}
actin filament-based movement	GO:0030048	1.235×10^{-2}

1 to 1 of 1 Page 1 of 1

GO:CC Term name	Term ID	p_{adj}
supramolecular fiber	GO:0099512	1.709×10^{-2}
supramolecular polymer	GO:0099081	1.763×10^{-2}
intracellular non-membrane-bounded organelle	GO:0043232	2.217×10^{-2}
non-membrane-bounded organelle	GO:0043228	2.239×10^{-2}

Genes: MYL1, PAXIP1, PPP1CA, SCN3B, TUBG1, WIPF1

Supplement Figure 1a: g:profiler gene set enrichment analysis of genes listed jointly in Tables 2 and 3.

GO:CC Term name	Term ID	p_{adj}
terminal cisterna	GO:0014802	2.875×10^{-3}
glycogen granule	GO:0042587	1.003×10^{-2}
PTW/PP1 phosphatase complex	GO:0072357	1.003×10^{-2}
protein-containing complex	GO:0032991	2.962×10^{-2}
protein phosphatase type 1 complex	GO:0000164	4.981×10^{-2}

1 to 5 of 5 Page 1 of 1

KEGG Term name	Term ID	p_{adj}
Long-term potentiation	KEGG:04720	3.081×10^{-2}

Genes: ADAM10, APBB1IP, CEBPB, CREBBP, EIF3F, INTS12, KPNA3, MEX3C, MRPL19, MYL1, PAXIP1, PCNX2, PPP1CA, PPP1CB, PPP2R3C, RAB2A, RAB31, RPL29, RTN2, RYR1, SCN3B, SIX4, SNRPD1, TMEM70, TUBG1, WIPF1, ZC3H15, SQSTM1

Supplement Figure 1b: g:profiler gene set enrichment analysis of genes listed in Table 3.

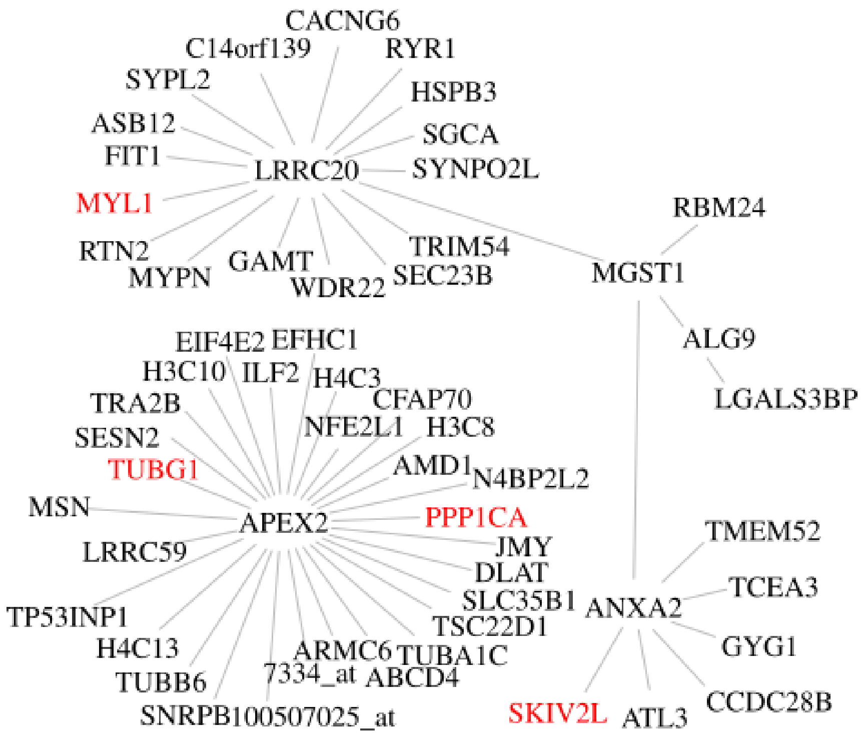

Supplement Figure 2a: Gene interactions observed in human. *The genes MYL1, PPP1CA, SKIV2L and TUBG1 are hub genes in WGCNA-defined modules from multiple species (cf. Figure 1, here shown in red). This graph was created iteratively with the red genes as a seed, then adding all the gene-gene interactions from WGCNA in human experiments originating from these red genes, and then transitively adding all the interacting genes of those. The resulting graph is highly interconnected before applying the minimum spanning tree algorithm. The genes interacting with hub genes across species (in red) then appear marginalized by the three human-only hub genes ANXA2, APEX2, LRRC20 and MGST1, given that the minimum spanning tree shows only the strongest correlations. ANXA2 is well described for a wide array of disease, i.e. cancer but also pulmonary fibrosis, and on a molecular level chimes in with vesicle fusion. APEX2 is a nuclease required for lymphocyte proliferation. LRRC20 is not yet described but known to interact with the also mostly undescribed TOM1 that once more is thought to be involved in intracellular trafficking and the E3 SUMO-protein ligase ZBED1. MGST1 is an enzyme located at the ER and mitochondria, a transferase of glutathione, an antioxidant.*

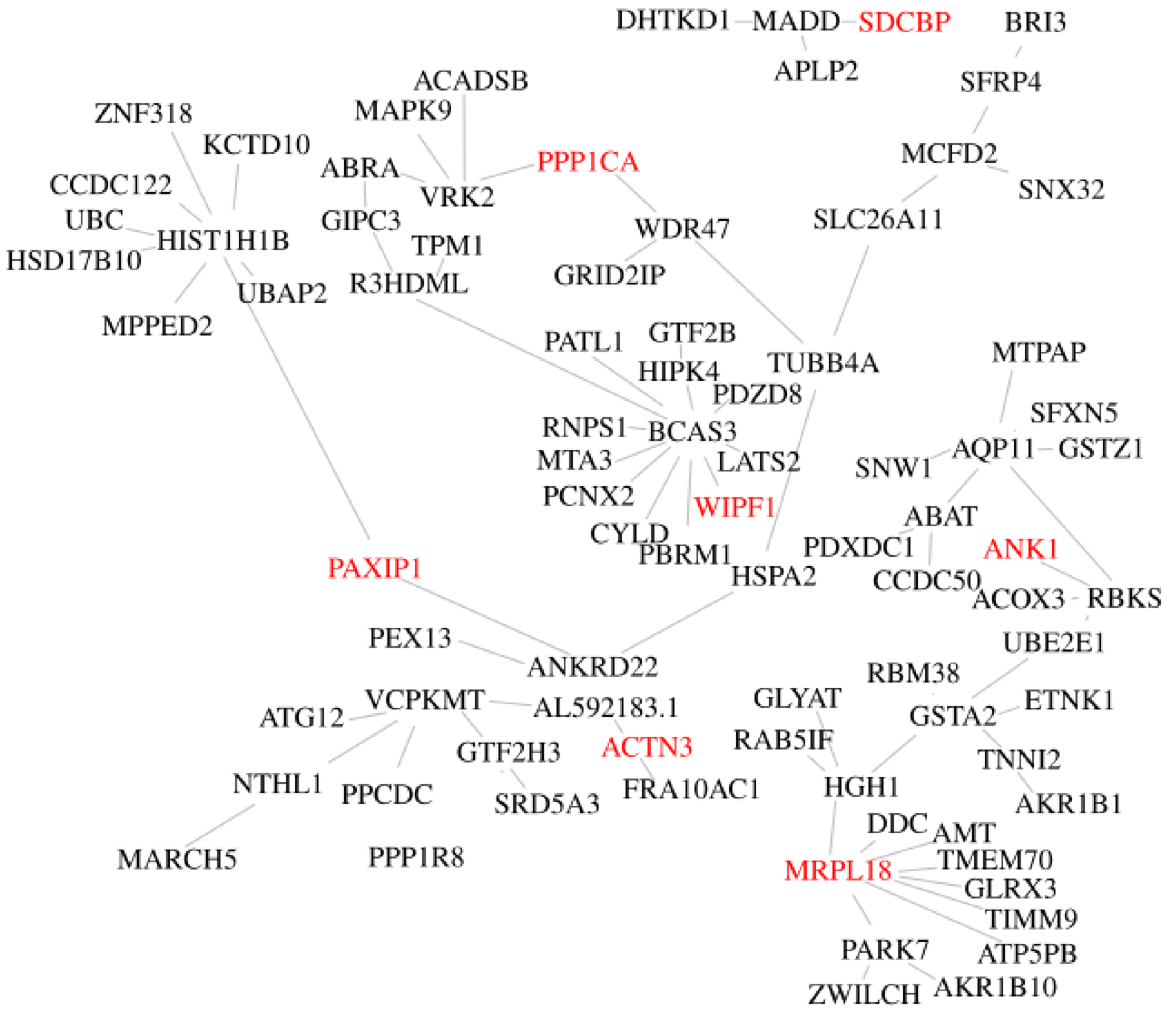

Supplement Figure 2b: *Gene interactions observed in worm. The figure was prepared analogously to Supplement Figure 2a from gene interactions observed in the worm. Gene names were mapped to human orthologs for an easier comparison between species.*

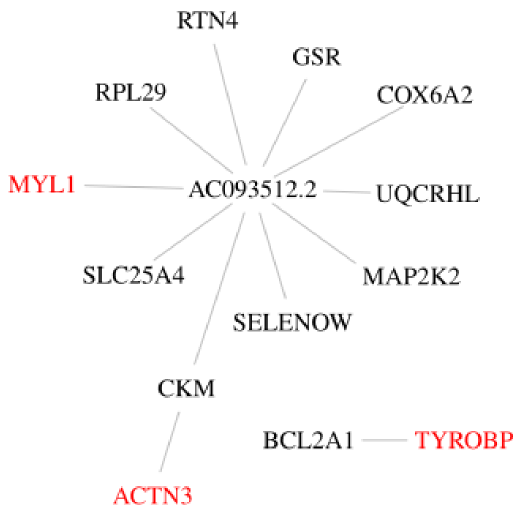

Supplement Figure 2c: Gene interactions observed in rat.

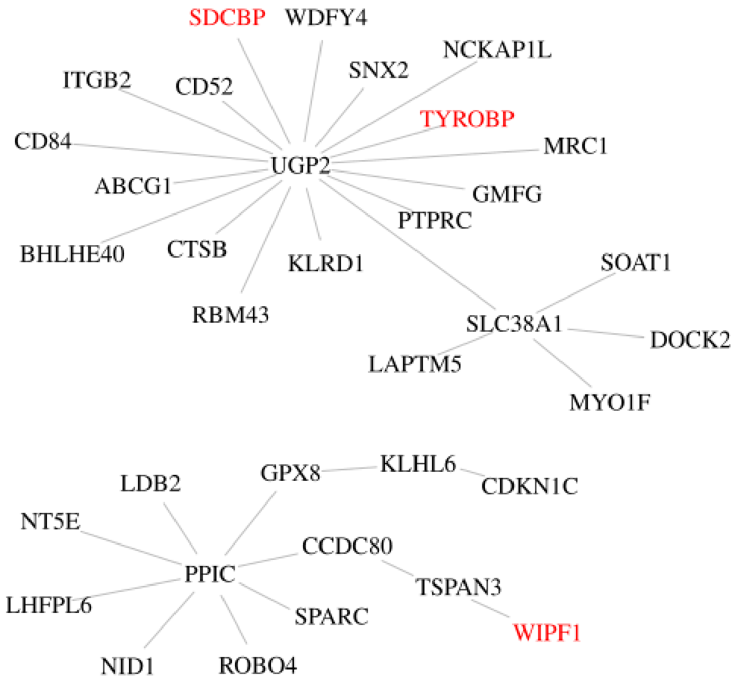

Supplement Figure 2d: Gene interactions observed in mouse.

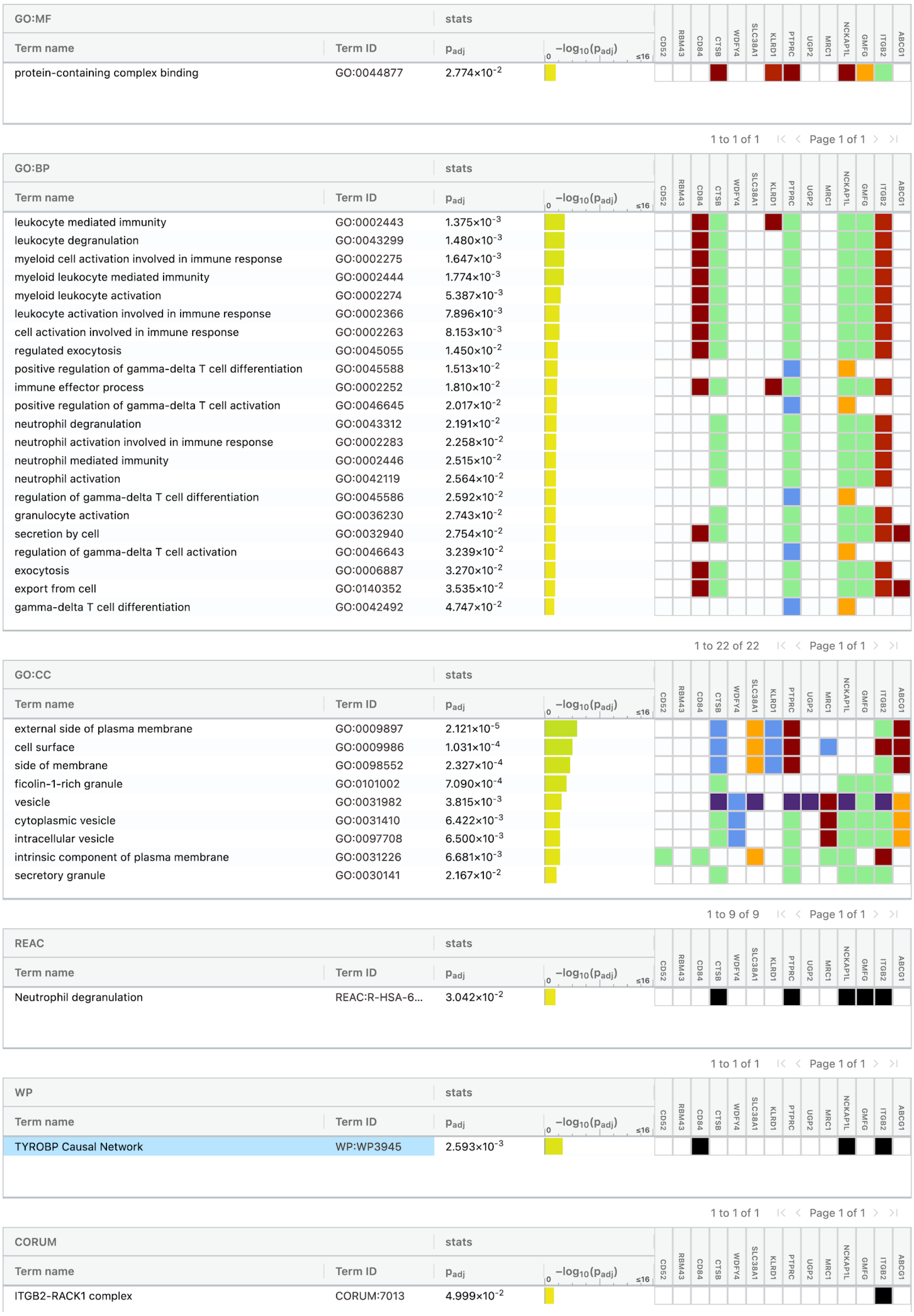

GO:MF Term name	Term ID	p_{adj}
protein-containing complex binding	GO:0044877	2.774×10^{-2}

1 to 1 of 1 Page 1 of 1

GO:BP Term name	Term ID	p_{adj}
leukocyte mediated immunity	GO:0002443	1.375×10^{-3}
leukocyte degranulation	GO:0043299	1.480×10^{-3}
myeloid cell activation involved in immune response	GO:0002275	1.647×10^{-3}
myeloid leukocyte mediated immunity	GO:0002444	1.774×10^{-3}
myeloid leukocyte activation	GO:0002274	5.387×10^{-3}
leukocyte activation involved in immune response	GO:0002366	7.896×10^{-3}
cell activation involved in immune response	GO:0002263	8.153×10^{-3}
regulated exocytosis	GO:0045055	1.450×10^{-2}
positive regulation of gamma-delta T cell differentiation	GO:0045588	1.513×10^{-2}
immune effector process	GO:0002252	1.810×10^{-2}
positive regulation of gamma-delta T cell activation	GO:0046645	2.017×10^{-2}
neutrophil degranulation	GO:0043312	2.191×10^{-2}
neutrophil activation involved in immune response	GO:0002283	2.258×10^{-2}
neutrophil mediated immunity	GO:0002446	2.515×10^{-2}
neutrophil activation	GO:0042119	2.564×10^{-2}
regulation of gamma-delta T cell differentiation	GO:0045586	2.592×10^{-2}
granulocyte activation	GO:0036230	2.743×10^{-2}
secretion by cell	GO:0032940	2.754×10^{-2}
regulation of gamma-delta T cell activation	GO:0046643	3.239×10^{-2}
exocytosis	GO:0006887	3.270×10^{-2}
export from cell	GO:0140352	3.535×10^{-2}
gamma-delta T cell differentiation	GO:0042492	4.747×10^{-2}

1 to 22 of 22 Page 1 of 1

GO:CC Term name	Term ID	p_{adj}
external side of plasma membrane	GO:0009897	2.121×10^{-5}
cell surface	GO:0009986	1.031×10^{-4}
side of membrane	GO:0098552	2.327×10^{-4}
ficolin-1-rich granule	GO:0101002	7.090×10^{-4}
vesicle	GO:0031982	3.815×10^{-3}
cytoplasmic vesicle	GO:0031410	6.422×10^{-3}
intracellular vesicle	GO:0097708	6.500×10^{-3}
intrinsic component of plasma membrane	GO:0031226	6.681×10^{-3}
secretory granule	GO:0030141	2.167×10^{-2}

1 to 9 of 9 Page 1 of 1

REAC Term name	Term ID	p_{adj}
Neutrophil degranulation	REAC:R-HSA-6...	3.042×10^{-2}

1 to 1 of 1 Page 1 of 1

WP Term name	Term ID	p_{adj}
TYROBP Causal Network	WP:WP3945	2.593×10^{-3}

1 to 1 of 1 Page 1 of 1

CORUM Term name	Term ID	p_{adj}
ITGB2-RACK1 complex	CORUM:7013	4.999×10^{-2}

Supplement Figure 3a: g:Profiler GSEA on genes connecting hub genes TYROBP and SDCBP.

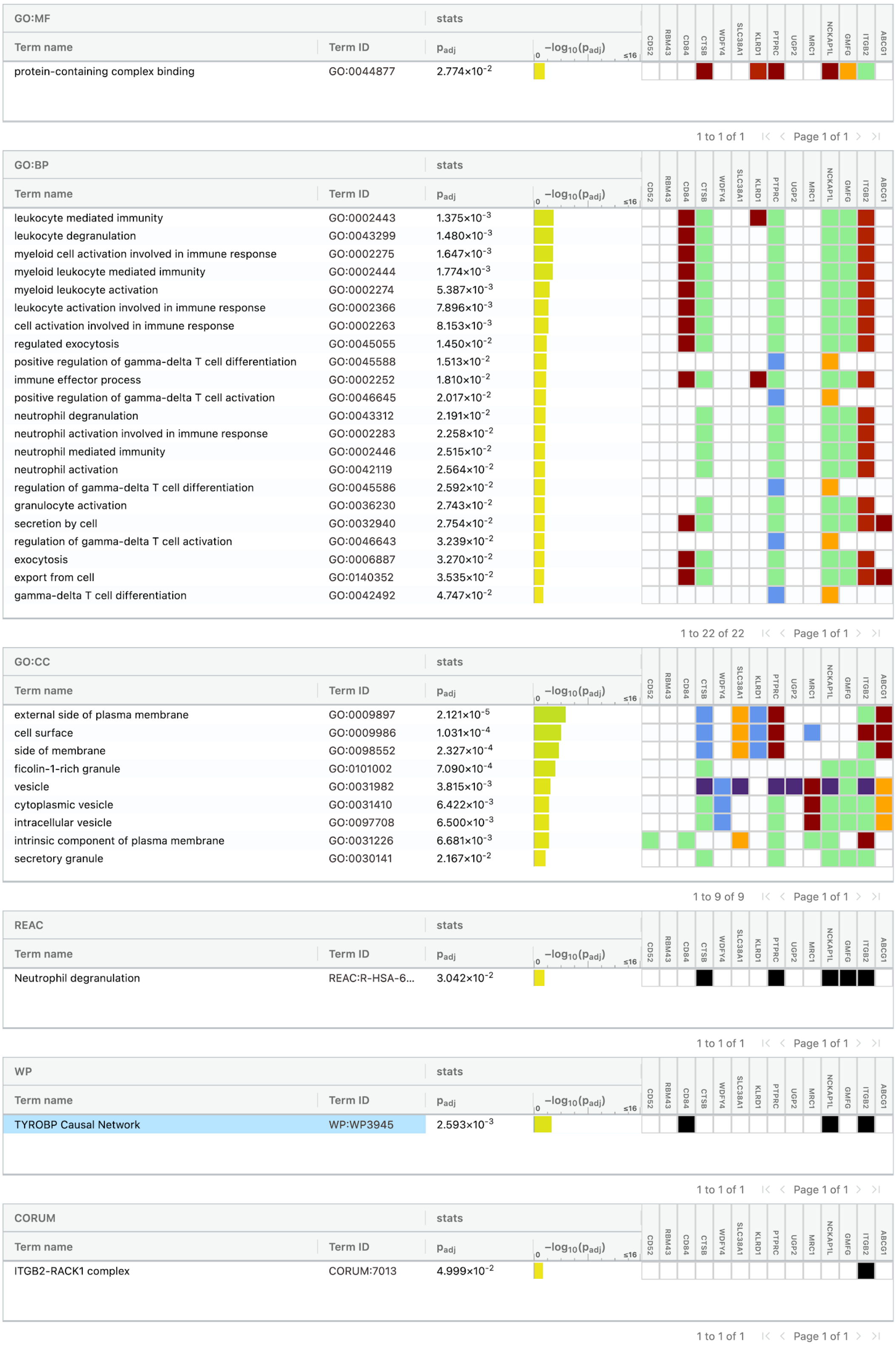

Supplement Figure 3b: g:Profiler GSEA on genes connecting hub genes MYL1 and SKIV2L.

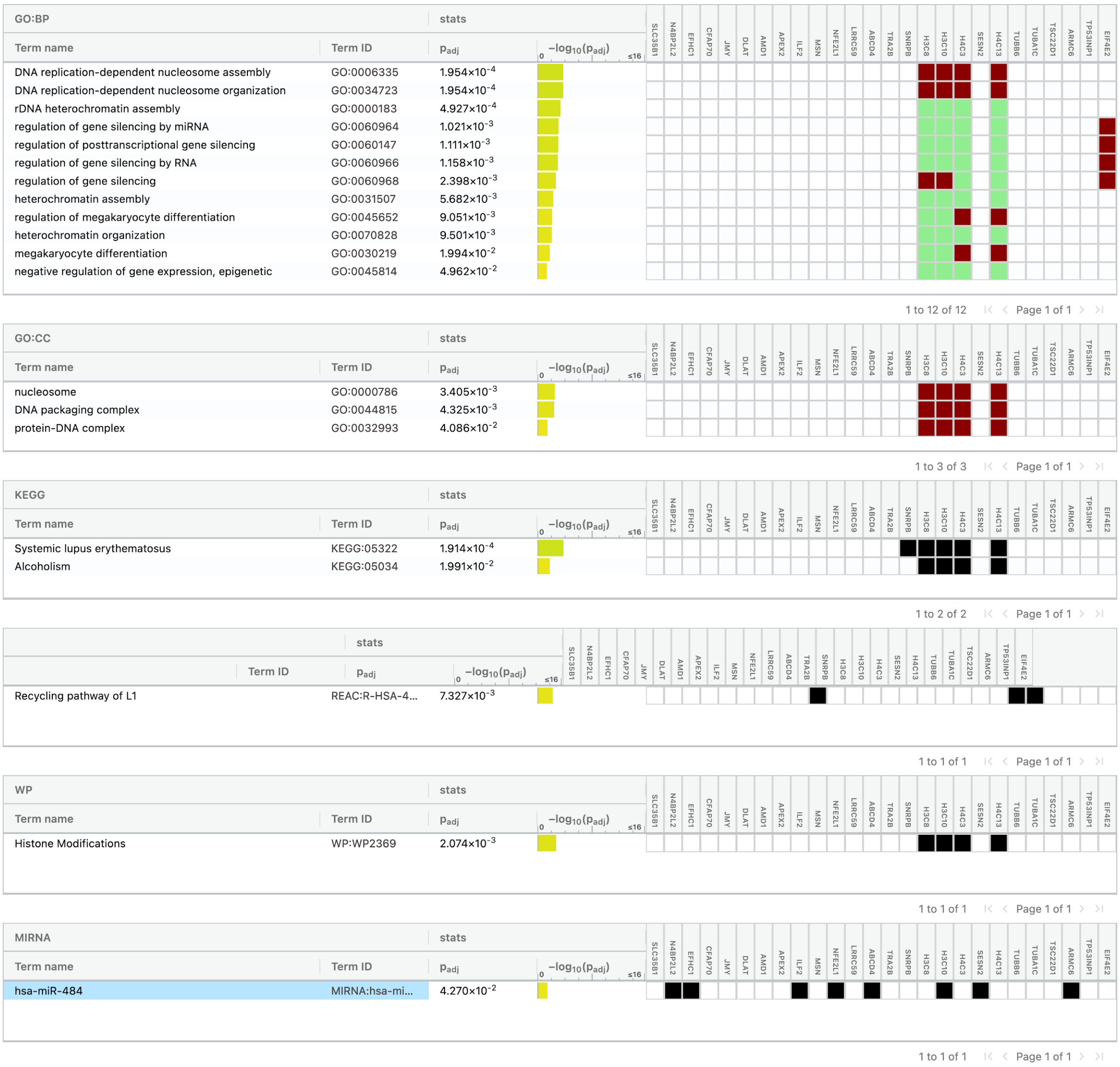

Supplement Figure 3c: g:Profiler GSEA on genes connecting hub genes TUBG1 and PPP1CA.

Tables

Supplement Table 1: List of ArrayExpress/GEO files used as input in our study. *This table provides an overview of the transcriptomics experiments that were retrieved for this study. Each experiment was processed by a regular WGCNA workflow with unsigned correlation. Interactions were collected for the 30 most connected (hub) genes in each module. The column Modules lists the number of modules found for the experiment that feature an eigengene that correlates (with P<0.05) with the samples' health phenotype score. Within each module, only interactions with an adjacency value above the 95th percentile of an experiment were considered. The rightmost column lists the number of different hub genes that are paired in any of these interactions. Numbers in parentheses give the number of genes/interactions that could be mapped to ortholog genes in the human; for human data, the number of orthologs in worm are shown. For an interaction, both of the paired genes need to have orthologs assigned; otherwise they were not considered for the count.*

Accession	Treatment groups	Description	Number of (with orthologs)			
			Samples [total[1] / per group]	Modules	Inter-actions	Genes
**a) Mouse experiments on healthspan**						
E-GEOD-19102 (array)	i) high-fat diet + 100mg/kg SRT1720, ii) high-fat diet + 30mg/kg SRT1720, iii) high-fat diet, iv) standard AIN-93G diet	An activator of Sirt1, SRT1720, extends healthspan and lifespan in diet-induced obese mice; samples: liver tissue from strain C57BL/6J	12/3	10	324 (182)	28 (20)
E-GEOD-34773 (array)	i) PGC-1a skeletal muscle specific knockout, ii) PGC-1a skeletal muscle specific knockout + CR, iii) WT, iv) WT + CR	PGC-1a is a transcriptional coactivator that regulates mitochondrial function and is induced by CR; PGC-1a mediates mitochondrial, but not metabolic, changes during calorie restriction (Finley *et al.*, 2012)	26/6-7	12	0 (0)	0 (0)
E-GEOD-40936 (array)	i) ad libitum diet, muscle tissue, ii) ad libitum diet + 0.1% w/w metformin, muscle tissue, iii) 40% calorically restricted diet, muscle tissue, iv) ad libitum diet, liver tissue, v) ad libitum diet + 0.1% w/w metformin, liver tissue, vi) 40% calorically restricted diet, liver tissue	Chronic treatment with low dose of metformin (0.1% w/w in diet) starting at one year of age extends health and lifespan in male mice, while a higher dose (1% w/w) was toxic	30/5	1	434 (0)	29 (23)
E-GEOD-49000 (array)	i) standard diet, muscle tissue, ii) standard diet liver tissue, iii) standard diet + SRT2104, muscle tissue, iv) standard diet + SRT2104, liver tissue, v) 40% Caloric restriction, muscle tissue, vi) 40% Caloric restriction, liver tissue	SRT2104, a synthetic small molecule activator of SIRT1, extends survival of male mice on a standard diet and preserves bone and muscle mass (Mercken *et al.*, 2014)	30/5	no modules found		

E-GEOD-54853 (RNA-seq)	i) 10 g/kg D-Glucoseamine, ii) untreated	D-Glucoseamine mimics a ketogenic diet and extends lifespan of aging C57BL/6 mice	12/6	1	435 (0)	30 (19)
E-GEOD-55272 (array)	i) WT, 5 months, liver tissue, ii) WT, 24 months, liver tissue, iii) WT, 5 months, muscle tissue, iv) WT, 24 months, muscle tissue, v) WT, 5 months, adipose tissue, vi) WT, 24 months, adipose tissue, vii) Myc+/- , 5 months, liver tissue, viii) Myc+/- , 24 months, liver tissue, ix) Myc+/- , 5 months, muscle tissue, x) Myc+/- 24 months, muscle tissue, xi) Myc+/- , 5 months, adipose tissue, xii) Myc+/- , 24 months, adipose tissue	The pleiotropic transcription factor MYC is a proto-oncogene and Myc+/- heterozygous mice have extended lifespan and improved healthspan	36/3	6	567 (377)	79 (64)
E-MTAB-6578 (RNA-seq)	female heart tissue: i) 10 weeks, HGPS-/- mutants, ii) 10 weeks, HGPS-/-; NAT10-/- double mutants, iii) 10 weeks, NAT10-/- mutants, iv) 10 weeks, HGPS-/- mutants + 100 mg/kg remodelin, v) 104 weeks, WT, vi) 10 weeks, WT	Targeting NAT10 enhances healthspan and lifespan in a mouse model of human accelerated aging syndrome (Hutchinson-Gilford progeria syndrome)	12/2	1	0 (0)	0 (0)

b) Rat experiments on healthspan

E-GEOD-38062 (array)	i) fed ad libitum, ii) fed 40% CR	Muscle specimens of rats following a calorie restriction (40%) diet vs ad libitum fed rats; age: from 2-27 months (Mercken *et al.*, 2013)	10/5	8	467 (81)	57 (24)

c) Human experiments on healthspan

E-GEOD-38012 (array)	i) middle-aged humans under CR diet, ii) middle-aged humans under western diet	Skeletal muscle specimens of humans following a calorie restriction diet vs humans following a Western diet	25/10-15	2	405 (324)	28 (25)
E-GEOD-66236 (array)	i) adipocytes proliferating in culture, ii) adipocytes in gamma-irradiation induced senescence	Difference between senescent and non-senescent cells in order to develop senolytic drugs	16/8	1	435 (276)	30 (24)

d) Worm experiments on healthspan

E-GEOD-8696 (array)	i) 4 µM, ii) 20 µM, & iii) 500 µM hemin	To understand heme homeostasis, genes transcriptionally regulated by heme should be identified	9/3	4	22 (0)	20 (7)

		(Rajagopal *et al.*, 2008)				
E-GEOD-9246 (array)	i) *slr-2*(ku297) mutants, ii) WT	Transcription profiling of *C. elegans slr-2* (C2H2 Zn-finger protein) mutants at L1 stage	6/3	1	434 (104)	29 (14)
E-GEOD-9301 (array)	i) 99% O2 (oxidative stress), ii) 99% O2 + *skn-1* RNAi, iii) untreated	*C. elegans* treated with oxidative stress in absence and presence of the transcription factor SKN-1, which is involved in response to oxidative stress	11/3-4		no modules found	
E-GEOD-21531 (array)	i) *unc-32; glp-1* double mutants (excess proliferation), ii) *unc-32* mutants	Analysis of germ cell proliferation in *unc-32*(e189);*glp-1*(oz112gf) double mutants (excess germ cell proliferation) compared to *unc-32*(e189) mutants as control (Waters *et al.*, 2010)	8/4		no modules found	
E-GEOD-30505 (array)	i) L3, WT, EV, ii) L3, WT, *ash-2* RNAi, iii) L3, *glp-1*(e2141ts) mutants, EV, iv) L3, *glp-1*(e2141ts) mutants, *ash-2* RNAi, v) day 8, WT, EV, vi) day 8, WT, *ash-2* RNAi, vii) day 8, *glp-1*(e2141ts) mutants, EV, viii) day 8, *glp-1*(e2141ts) mutants, *ash-2* RNAi	The ASH-2 trithorax complex trimethylates histone H3 at lysine 4 (H3K4); *ash-2* knock-down increases lifespan in a germline dependent manner. *ash-2* knock-down was compared in young and old as well as in WT and germline deficient (*glp-1*) mutants (Greer et al., 2010)	23/2-3	4	854 (146)	60 (20)
E-GEOD-32031 (array)	i) *nhr-23* RNAi, ii) untreated	Inhibition of *nhr-32*, important for growth and molting, in L2 larvae	6/3	1	124 (2)	27 (5)
E-GEOD-32339 (array)	i) L3 WT, ii) L3 *nep-1* mutants, iii) adult WT, iv) adult *nep-1* mutants	Comparison between wild-type and *nep-1* (homologue of human ECE1 (endothelin-converting enzyme 1) mutant strain	10/2-3	3	867 (173)	57 (25)
E-GEOD-35939 (array)	embryonic touch-receptor cells with i) mutant Huntingtin (128Q) vs normal Huntingtin (19Q), ii) normal Huntingtin (19Q) vs GFP only	Comparison of purified touch receptor neurons expressing mutant Huntingtin N-terminal fragment (expanded polyGlutamine) with normal Huntingtin N-terminal fragment	12/6		no modules found	
E-GEOD-36494 (RNA-seq)	i) WT, ii) WT + TAP (tobacco acid pyrophosphatase), iii) *rde-10* mutant, iv) *rde-10* mutant + TAP, v) *rde-11* mutant, vi) *rde-11* mutant + TAP)	The RDE-10/RDE-11 complex triggers RNAi induced mRNA degradation by association with target mRNA in *C. elegans* (Yang et al., 2012)	12/2		no modules found	
E-GEOD-38877 (array)	centrifuged at i) 1g, ii) 5g, iii) 10g, iv) 15g	Worms spun in centrifuge at elevated g values	18/3-9		no modules found	
E-GEOD-40459 (array)	i) *rsd-2* (yp10) mutants, 25 °C, late generation, ii) *rsd-2* (yp10) mutants, 25 °C, early generation, iii) *rsd-2* (pk3307) mutants, 25°C, late generation, iv) *rsd-2* (pk3307) mutants, 25°C, early generation, v) *rsd-6* (pk3300) mutants, 25°C, late generation, vi) *rsd-6* (pk3300) mutants, 25°C, early generation,vii) *rsd-2* (yp10) mutants, 20 °C, late	Effects of transgeneration aging studied in early and late generation *rsd-2* and *rsd-6* mutants at the restrictive temperature of 25°C and the permissive temperature of 20°C	36/3		no modules found	

	generation, viii) *rsd-2* (yp10) mutants, 20 °C, early generation, ix) *rsd-2* (pk3307) mutants, 20°C, late generation, x) *rsd-2* (pk3307) mutants, 20°C, early generation, xi) *rsd-6* (pk3300) mutants, 20°C, late generation, xii) *rsd-6* (pk3300) mutants, 20°C, early generation					
E-GEOD-42192 (array)	i) fed with E.coli OP50 for 3 days or ii) 10 days, iii) fed with Lactobacillus rhamnosus CNCM I-3690 for 3 days or iv) 10 days, v) fed with Lactobacillus rhamnosus CNCM I-4317 for 3 days or vi) 10 days	The lactic acid bacteria *Lactobacillus rhamnosus* (CNCM I-3690) increase worm's lifespan by antioxidative actions	18/3	2	868 (71)	58 (15)
E-GEOD-43864 (array)	i) *nhr-114* RNAi, ii) *glp-1*(q224ts) mutants, iii) WT untreated, iv) WT + tryptophan	The nuclear receptor *nhr-114*/HNF4 protects germline stem cells from dietary metabolites. The downregulation of *nhr-114* results in germline defects and sterility, which depends on tryptophan. Sterile *glp-1* mutants are used for comparison. animals	12/3	9	1287 (263)	73 (31)
E-GEOD-46051 (RNA-seq)	i) day 1, ii) day 1 + 100 nM rotenone, iii) day 5, iv) day 5 + 100 nM rotenone, v) day 10, vi) day 10 + 100 nM rotenone, vii) day 20, viii) day 20 + 100 nM rotenone	Deep sequencing of endogenous mRNA from Caenorhabditis elegans in the presence and absence of rotenone at 4 different time points (Schmeisser *et al.*, 2013)	22/2-3	21	3123 (474)	264 (74)
E-GEOD-51502 (array)	i) genetically activated beta-catenin, ii) WT	Use of an activated beta-catenin to identify Wnt/beta-catenin pathway target genes (Jackson *et al.*, 2014)	6/3	3	1302 (112)	86 (27)
E-GEOD-52340 (array)	i) WT, ii) *rsks-1* mutants, iii) *daf-2* mutants, iv) daf-2;rsks-1 double mutants, v) daf-16;daf-2;rsks-1 triple mutants	Synergistic lifespan extension in daf-2;rsks-1 double mutants requires DAF-16 and the germline was identified as the key tissue for this synergistic longevity (Chen *et al.*, 2013)	47/9-10		no modules found	
E-GEOD-54853 (RNA-seq)	i) 100 µM D-Glucosamine, ii) untreated	D-Glucosamine extends C. elegans lifespan by impairing glucose metabolism to activate AMP-activated protein kinase	12/6	2	870 (0)	60 (20)
E-GEOD-57739 (RNA-seq)	i) WT + standard food (OP50), ii) WT + *S. aureus*, iii) *hlh-30* mutants + OP50, iv) *hlh-30* mutants + *S. aureus*	HLH-30/TFEB is a transcription factor in the host response to infections and regulates the transcription of cytoprotective and antimicrobial genes (Visvikis *et al.*, 2014)	8/2		685 (55)	90 (28)
E-GEOD-85342 (array)	i) 5-fluorouracil, ii) DMSO	Treatment with 5-fluorouracil inhibits growth of *P. aeruginosa* and reduces pyoverdine biosynthesis	6/3		no modules found	
E-MEXP-479 (array)	i) fed with standard food for 12h or ii) 24h, iii) fed with *Drechmeria coniospora* for	Exposure to the fungal pathogen Drechmeria coniospora for 12 and 24 hours (Pujol *et al.*, 2008)	64/16		no modules found	

	12h or iv) 24h					
E-MTAB-1333 (array)	pash-1(mj100) mutants at restrictive temperature (25 °C) for i) 0, ii) 6, iii) 12 or iv) 24 hours, pash-1(mj100); pash-1::gfp mutants at 25 °C for v) 0, vi) 6, vii) 12 or viii) 24 hours	Reversible inactivation of miRNA synthesis via DGCR8/pash-1 conditional mutants (Lehrbach et al., 2012)	24/3	6	1715 (440)	119 (58)
E-MEXP-1808 (array)	i) strain DR1350, ii) strain DR1350 + dauer pheromone, iii) WT N2, iv) WT N2 + dauer pheromone	Wild type isolates treated with dauer larva-inducing pheromone (Harvey et al., 2009)	12/3		no modules found	
E-MEXP-1810 (array)	i) strain RIL-14, ii) strain RIL-14 + dauer pheromone, iii) strain RIL-17, iv) strain RIL-17 + dauer pheromone	Wild type isolates treated with dauer larva-inducing pheromone	12/3	6	261 (108)	65 (37)

[1] total: total samples in this experiment; per group: samples per treatment-group (biological replicates)

Supplement Table 2: Genes correlating with eigengene representation (module membership) *of those modules that are correlating with the health score. The table lists all genes that appear in at least two experiments among the top 30 and a P value below 0.05. If the expression of the gene correlates positively with the health score then the gene is tagged as positive. The columns "cor" and "P val" list the values as determined by WGCNA. A negative correlation with a positive tag indicates that eigengene of the module is negatively correlated with the health score.*

Gene	health	cor	P val	Experiment	module
0610006I08Rik	negative	0.907	4.64e-05	E-GEOD-19102	darkmagenta
0610006I08Rik	negative	0.942	4.57e-06	E-GEOD-19102	skyblue3
2010107E04Rik	positive	0.951	6.24e-19	E-GEOD-55272	black
2010107E04Rik	positive	-0.947	2.10e-18	E-GEOD-55272	magenta
ABAT	positive	0.988	2.21e-18	E-GEOD-30505	blue
ABAT	positive	-0.980	1.42e-12	E-GEOD-42192	turquoise
ABCG1	negative	-0.900	6.76e-05	E-GEOD-19102	sienna2
ABCG1	negative	0.974	1.68e-23	E-GEOD-55272	red
ABRA	negative	0.996	4.41e-12	E-GEOD-43864	brown
ABRA	negative	0.989	2.90e-06	E-GEOD-57739	turquoise
ABRA	positive	-0.968	1.12e-14	E-MTAB-1333	blue
abu-14	positive	0.993	5.68e-21	E-GEOD-30505	turquoise
abu-14	positive	0.999	7.55e-07	E-GEOD-9246	turquoise
AC068831.7	negative	-0.868	2.48e-04	E-GEOD-19102	yellow3
AC068831.7	negative	0.986	1.28e-18	E-MTAB-1333	turquoise
AC106774.4	negative	-0.873	2.13e-04	E-MEXP-1810	plum1
AC106774.4	negative	0.938	6.73e-06	E-MEXP-1810	royalblue
ADAM10	negative	-0.871	7.20e-09	E-GEOD-34773	greenyellow
ADAM10	positive	0.998	4.87e-13	E-GEOD-43864	turquoise
AGO1	negative	-0.995	2.14e-09	E-GEOD-32339	blue
AGO1	negative	-0.879	1.53e-08	E-MTAB-1333	black
ain-1	negative	0.921	4.24e-04	E-GEOD-8696	cyan
ain-1	negative	0.965	2.56e-05	E-GEOD-8696	royalblue
APBB1IP	positive	0.987	2.78e-09	E-GEOD-54853-CEL	yellow
APBB1IP	negative	0.970	1.68e-07	E-MTA-B6578	blue
AQP11	positive	0.997	2.57e-24	E-GEOD-30505	blue
AQP11	negative	0.951	1.98e-06	E-MEXP-1810	brown
ARMC1	positive	-0.850	3.97e-08	E-GEOD-34773	darkgrey
ARMC1	positive	0.874	5.48e-09	E-GEOD-34773	greenyellow
asns-2	negative	-0.993	7.01e-05	E-GEOD-51502	black
asns-2	positive	0.984	8.21e-09	EGEOD54853-CEL	brown
ATP6V1B2	negative	0.978	4.00e-08	E-GEOD-43864	paleturquoise
ATP6V1B2	negative	0.967	2.76e-07	E-GEOD-43864	paleturquoise
ATP6V1B2	positive	0.999	2.07e-06	E-GEOD-9246	turquoise
B2M	negative	0.927	1.03e-11	E-GEOD-34773	lightyellow
B2M	negative	0.922	2.13e-11	E-GEOD-34773	lightyellow
B2M	negative	0.975	6.82e-08	EMTAB6578	blue
C02E7.6	positive	0.993	4.20e-21	E-GEOD-30505	turquoise
C02E7.6	positive	0.990	9.63e-10	E-GEOD-43864	yellow
C09B9.2	negative	0.990	2.56e-06	E-GEOD-57739	turquoise
C09B9.2	positive	0.989	1.36e-19	E-MTAB-1333	yellow
C26F1.1	positive	0.989	1.13e-09	E-GEOD-43864	yellow
C26F1.1	negative	0.982	4.60e-04	E-GEOD-51502	turquoise
C46F2.1	negative	-0.869	2.45e-04	E-MEXP-1810	plum1
C46F2.1	negative	0.921	2.15e-05	E-MEXP-1810	royalblue

C46G7.1	positive	-0.979	1.62e-12	E-GEOD-42192	turquoise
C46G7.1	negative	0.928	8.98e-04	E-GEOD-57739	darkgreen
CAD	negative	-0.875	1.93e-04	E-MEXP-1810	plum1
CAD	negative	0.902	6.02e-05	E-MEXP-1810	royalblue
CD151	negative	0.934	8.51e-06	E-GEOD-19102	darkmagenta
CD151	negative	-0.905	2.28e-10	E-GEOD-34773	tan
CEBPB	positive	0.940	1.65e-04	E-GEOD-38062	salmon
CEBPB	negative	0.812	5.27e-08	E-GEOD-40936	grey
CLIC5	negative	0.842	6.00e-04	E-GEOD-19102	green3
CLIC5	negative	-0.883	2.42e-09	E-GEOD-34773	greenyellow
comp-1	negative	0.991	2.09e-06	E-GEOD-57739	turquoise
comp-1	positive	0.991	9.50e-21	E-MTAB-1333	yellow
COX15	positive	-0.890	1.19e-09	E-GEOD-34773	darkgrey
COX15	positive	-0.879	3.61e-09	E-GEOD-34773	darkgrey
COX15	positive	0.923	1.79e-11	E-GEOD-34773	greenyellow
cpg-2	negative	-0.994	5.19e-09	E-GEOD-32339	blue
cpg-2	positive	0.961	3.76e-05	E-GEOD-8696	red
CRB1	negative	-0.958	1.85e-04	E-GEOD-57739	darkorange
CRB1	positive	0.989	1.48e-09	EGEOD54853-CEL	yellow
CREBBP	negative	0.953	1.96e-05	E-GEOD-32339	pink
CREBBP	negative	0.937	1.75e-12	E-GEOD-34773	red
CREBBP	negative	0.958	4.89e-05	E-GEOD-8696	purple
D2062.7	positive	-0.969	4.07e-11	E-GEOD-42192	blue
D2062.7	positive	0.985	1.99e-18	E-MTAB-1333	yellow
DCP2	negative	0.928	1.74e-10	E-GEOD-30505	black
DCP2	negative	-0.997	2.13e-10	E-GEOD-32339	blue
DDX4	positive	-0.998	3.10e-13	E-GEOD-43864	blue
DDX4	negative	-0.877	1.77e-04	E-MEXP-1810	lightcyan
DEPDC1B	negative	0.915	9.48e-10	E-GEOD-30505	black
DEPDC1B	positive	-0.934	2.28e-04	E-GEOD-8696	cyan
dnj-3	positive	0.940	5.49e-06	E-GEOD-43864	royalblue
dnj-3	negative	0.924	1.05e-03	E-GEOD-57739	darkgreen
dnj-3	negative	0.974	9.23e-16	E-MTAB-1333	brown
DRG1	negative	0.993	6.92e-05	E-GEOD-51502	turquoise
DRG1	negative	0.925	1.64e-05	E-MEXP-1810	brown
EEF1B2	negative	-0.866	2.71e-04	E-GEOD-19102	deeppink
EEF1B2	positive	0.948	1.93e-13	E-GEOD-34773	green
egg-2	positive	-0.998	8.73e-14	E-GEOD-43864	blue
egg-2	positive	-0.998	2.95e-13	E-GEOD-43864	blue
egg-2	positive	0.964	2.85e-05	E-GEOD-8696	red
EIF3F	positive	-0.929	7.24e-12	E-GEOD-34773	purple
EIF3F	positive	0.999	9.64e-07	E-GEOD-9246	turquoise
EMC3	negative	-0.915	5.49e-04	E-GEOD-38062	darkgreen
EMC3	negative	0.954	6.54e-05	E-GEOD-38062	plum1
F45D3.4	positive	0.935	8.05e-06	E-MEXP-1810	darkmagenta
F45D3.4	positive	-0.917	2.69e-05	E-MEXP-1810	royalblue
F55A3.2	positive	0.867	2.62e-04	E-MEXP-1810	darkmagenta
F55A3.2	positive	0.876	1.89e-04	E-MEXP-1810	darkolivegreen
FBLN7	negative	0.877	1.83e-04	E-GEOD-19102	green3
FBLN7	negative	0.909	1.37e-10	E-GEOD-34773	magenta
fkb-7	positive	-0.995	4.39e-05	E-GEOD-51502	turquoise
fkb-7	positive	0.965	1.05e-04	E-GEOD-57739	sienna3
flp-15	positive	0.959	8.29e-07	E-MEXP-1810	plum1
flp-15	positive	0.914	3.14e-05	E-MEXP-1810	plum1

flp-15	positive	-0.898	7.43e-05	E-MEXP-1810	royalblue
GMFG	negative	0.892	9.67e-05	E-GEOD-19102	darkmagenta
GMFG	negative	0.984	6.89e-27	E-GEOD-55272	red
GNG10	negative	0.927	1.40e-05	E-GEOD-19102	darkmagenta
GNG10	positive	0.959	3.66e-20	E-GEOD-55272	yellow
GPR142	negative	0.990	7.71e-10	E-GEOD-43864	lightgreen
GPR142	negative	0.979	3.46e-08	E-GEOD-43864	lightgreen
GPR142	negative	0.925	3.56e-04	E-GEOD-8696	cyan
grl-7	positive	0.992	4.32e-20	E-GEOD-30505	turquoise
grl-7	positive	0.993	1.22e-10	E-GEOD-43864	yellow
grl-7	positive	0.991	3.61e-10	E-GEOD-43864	yellow
grl-7	positive	0.991	3.91e-10	E-GEOD-43864	yellow
GSTA2	positive	0.992	3.59e-20	E-GEOD-30505	blue
GSTA2	negative	0.976	6.93e-06	E-GEOD-8696	purple
GSTA2	negative	0.953	1.73e-06	E-MEXP-1810	brown
H2-D1	negative	0.951	9.09e-14	E-GEOD-34773	lightyellow
H2-D1	negative	0.948	1.95e-13	E-GEOD-34773	lightyellow
H2-D1	negative	0.941	9.01e-13	E-GEOD-34773	lightyellow
H2-D1	negative	0.894	7.30e-10	E-GEOD-34773	lightyellow
H2-D1	negative	0.974	9.03e-08	EMTAB6578	blue
H42K12.3	positive	0.992	2.23e-20	E-GEOD-30505	turquoise
H42K12.3	positive	0.988	2.15e-04	E-GEOD-51502	blue
HACL1	positive	0.881	1.55e-04	E-MEXP-1810	plum1
HACL1	positive	-0.963	5.25e-07	E-MEXP-1810	royalblue
HACL1	positive	-0.908	4.46e-05	E-MEXP-1810	royalblue
HSDL2	positive	0.940	5.23e-04	E-GEOD-57739	darkorange
HSDL2	positive	0.949	2.39e-06	E-MEXP-1810	lightcyan
IHH	negative	0.933	2.34e-04	E-GEOD-8696	cyan
IHH	positive	0.999	2.32e-06	E-GEOD-9246	turquoise
INTS12	negative	-0.845	5.42e-04	E-GEOD-19102	yellow3
INTS12	positive	-0.980	2.32e-08	E-GEOD-43864	paleturquoise
K08C9.2	negative	0.997	8.50e-13	E-GEOD-43864	brown
K08C9.2	positive	0.986	1.88e-18	E-MTAB-1333	yellow
KPNA3	negative	0.932	4.18e-12	E-GEOD-34773	red
KPNA3	positive	0.999	2.92e-06	E-GEOD-9246	turquoise
let-2	positive	0.992	1.36e-20	E-GEOD-30505	blue
let-2	positive	-0.979	1.83e-12	E-GEOD-42192	turquoise
LIAS	positive	-0.921	2.67e-11	E-GEOD-34773	black
LIAS	positive	0.880	3.27e-09	E-GEOD-34773	tan
LIN28A	positive	-0.998	1.06e-13	E-GEOD-43864	blue
LIN28A	positive	-0.998	2.15e-13	E-GEOD-43864	blue
LIN28A	negative	-0.877	1.78e-04	E-MEXP-1810	darkolivegreen
MARCKS	negative	0.967	3.10e-07	E-GEOD-19102	blue
MARCKS	negative	0.918	4.08e-11	E-GEOD-34773	magenta
MEX3C	negative	-0.994	7.28e-09	E-GEOD-32339	blue
MEX3C	negative	0.948	6.71e-13	E-GEOD-38012	yellow
mig-6	positive	-0.978	2.21e-12	E-GEOD-42192	turquoise
mig-6	positive	0.984	8.04e-09	EGEOD54853-CEL	yellow
MRAS	positive	0.989	5.85e-19	E-GEOD-30505	blue
MRAS	positive	-0.965	3.01e-14	E-MTAB-1333	blue
MRPL13	positive	-0.886	1.74e-09	E-GEOD-34773	darkgrey
MRPL13	positive	-0.878	3.75e-09	E-GEOD-34773	darkgrey
MRPL13	positive	0.867	9.86e-09	E-GEOD-34773	greenyellow
MRPL19	positive	-0.869	8.64e-09	E-GEOD-34773	darkgrey

MRPL19	negative	0.951	1.96e-06	E-MEXP-1810	brown
MRPL30	negative	0.888	1.13e-04	E-GEOD-19102	skyblue3
MRPL30	positive	0.939	2.45e-17	E-GEOD-55272	black
MTPAP	negative	-0.992	3.65e-20	E-GEOD-30505	blue
MTPAP	negative	-0.995	2.69e-09	E-GEOD-32339	blue
Myeov2	positive	0.931	1.71e-16	E-GEOD-55272	black
Myeov2	positive	-0.976	5.77e-24	E-GEOD-55272	magenta
MYL1	positive	-0.996	3.24e-25	E-GEOD-38012	turquoise
MYL1	positive	0.985	1.49e-06	E-GEOD-38062	tan
MYL7	negative	0.895	8.28e-05	E-GEOD-19102	skyblue3
MYL7	negative	0.901	6.40e-05	EGEOD54853-MMU	darkturquoise
NDUFS1	positive	0.862	3.07e-04	E-GEOD-19102	coral1
NDUFS1	positive	0.877	1.77e-04	E-GEOD-19102	lightsteelblue1
NR1H2	positive	0.983	1.22e-08	E-GEOD-43864	darkorange
NR1H2	negative	-0.909	1.75e-03	E-GEOD-57739	sienna3
NR1H2	positive	0.883	1.42e-04	E-MEXP-1810	darkolivegreen
NR1H2	positive	0.851	4.51e-04	E-MEXP-1810	darkolivegreen
NR1H2	positive	0.894	3.95e-09	E-MTAB-1333	black
NRBP1	negative	0.855	2.64e-08	E-GEOD-34773	darkgrey
NRBP1	negative	0.969	4.13e-22	E-GEOD-55272	pink
Pabpc1	positive	-0.887	1.21e-04	E-GEOD-19102	darkmagenta
Pabpc1	negative	0.969	2.22e-07	EMTAB6578	blue
PAXIP1	positive	0.890	1.03e-04	E-GEOD-19102	deeppink
PAXIP1	positive	0.998	2.67e-13	E-GEOD-43864	turquoise
PCNX2	negative	0.962	7.78e-14	E-MTAB-1333	blue
PCNX2	negative	0.907	4.66e-05	EGEOD54853-MMU	darkturquoise
PDCD6	negative	-0.959	8.71e-07	E-GEOD-43864	royalblue
PDCD6	negative	0.928	6.50e-11	E-MTAB-1333	green
PFAS	positive	-0.858	2.13e-08	E-GEOD-34773	darkgrey
PFAS	positive	0.941	8.58e-13	E-GEOD-34773	greenyellow
PPIA	positive	-0.975	6.83e-12	E-GEOD-42192	blue
PPIA	negative	0.918	2.52e-05	E-MEXP-1810	royalblue
PPIC	negative	0.981	1.67e-08	E-GEOD-19102	blue
PPIC	negative	0.981	1.97e-08	E-GEOD-19102	blue
PPIC	negative	0.917	4.55e-11	E-GEOD-34773	magenta
PPP1CA	positive	0.987	2.48e-04	E-GEOD-51502	black
PPP1CA	negative	0.991	1.68e-06	E-GEOD-57739	turquoise
PPP1CA	positive	0.993	2.02e-14	E-GEOD-66236	turquoise
PPP1CA	positive	0.989	9.26e-20	E-MTAB-1333	yellow
PPP1CB	positive	-0.979	4.67e-06	E-GEOD-38062	turquoise
PPP1CB	positive	0.999	1.18e-06	E-GEOD-9246	turquoise
PPP2R3C	negative	0.944	1.38e-12	E-GEOD-38012	yellow
PPP2R3C	negative	0.966	1.31e-21	E-GEOD-55272	pink
pqn-32	positive	0.993	3.79e-21	E-GEOD-30505	turquoise
pqn-32	positive	0.993	1.60e-10	E-GEOD-43864	yellow
pqn-32	positive	0.989	1.27e-09	E-GEOD-43864	yellow
PSMD4	positive	0.932	9.87e-06	E-GEOD-19102	lightpink2
PSMD4	negative	0.848	4.59e-08	E-GEOD-34773	darkgrey
ptp-2	negative	-0.937	7.07e-06	E-MEXP-1810	plum1
ptp-2	negative	0.932	1.03e-05	E-MEXP-1810	royalblue
PUM1	negative	-0.995	3.07e-09	E-GEOD-32339	blue
PUM1	negative	-0.995	3.51e-09	E-GEOD-32339	blue
PUM1	positive	0.986	9.40e-07	E-GEOD-8696	red
R3HDML	negative	-0.898	7.34e-05	E-MEXP-1810	plum1

R3HDML	negative	0.928	1.33e-05	E-MEXP-1810	royalblue
R3HDML	negative	0.916	2.80e-05	E-MEXP-1810	royalblue
RAB2A	negative	0.965	6.41e-06	E-GEOD-32339	pink
RAB2A	negative	0.960	2.47e-20	E-GEOD-55272	pink
RAB31	negative	0.952	1.77e-06	E-GEOD-19102	blue
RAB31	negative	0.971	1.37e-05	E-GEOD-38062	turquoise
RAD17	negative	0.994	5.90e-09	E-GEOD-32339	turquoise
RAD17	negative	0.982	1.90e-17	E-MTAB-1333	turquoise
RGL1	negative	0.956	1.25e-06	E-GEOD-19102	blue
RGL1	positive	-0.687	2.76e-05	E-GEOD-40936	grey
RMDN3	positive	-0.971	2.12e-11	E-GEOD-42192	blue
RMDN3	negative	0.999	3.97e-14	E-GEOD-43864	brown
RMDN3	negative	0.996	4.43e-12	E-GEOD-43864	brown
RMDN3	positive	0.967	2.03e-05	E-GEOD-8696	red
RPL11	positive	0.929	7.00e-12	E-GEOD-34773	green
RPL11	positive	0.963	7.42e-21	E-GEOD-55272	yellow
RPL13A	positive	0.936	2.43e-12	E-GEOD-34773	green
RPL13A	positive	-0.959	1.11e-14	E-GEOD-34773	purple
RPL23A	positive	0.934	3.34e-12	E-GEOD-34773	green
RPL23A	positive	0.929	7.50e-12	E-GEOD-34773	green
RPL23A	positive	-0.966	1.56e-15	E-GEOD-34773	purple
RPL23A	positive	-0.939	1.26e-12	E-GEOD-34773	purple
RPL23A	positive	-0.934	3.44e-12	E-GEOD-34773	purple
RPL27	positive	0.930	6.54e-12	E-GEOD-34773	green
RPL27	positive	-0.963	4.04e-15	E-GEOD-34773	purple
Rpl27a	positive	0.935	2.52e-12	E-GEOD-34773	green
Rpl27a	positive	0.965	2.64e-21	E-GEOD-55272	yellow
RPL29	positive	0.940	1.09e-12	E-GEOD-34773	green
RPL29	positive	0.976	7.35e-06	E-GEOD-38062	tan
RPS12	positive	0.942	7.63e-13	E-GEOD-34773	green
RPS12	positive	-0.926	1.28e-11	E-GEOD-34773	purple
RPS21	positive	0.954	4.18e-14	E-GEOD-34773	green
RPS21	positive	0.937	1.87e-12	E-GEOD-34773	green
RPS21	positive	-0.923	1.82e-11	E-GEOD-34773	purple
Rps25	positive	0.960	1.00e-14	E-GEOD-34773	green
Rps25	positive	-0.927	1.02e-11	E-GEOD-34773	purple
Rps29	positive	0.935	2.90e-12	E-GEOD-34773	green
Rps29	positive	-0.954	4.47e-14	E-GEOD-34773	purple
RPS7	negative	-0.905	5.22e-05	E-GEOD-19102	deeppink
RPS7	positive	-0.923	1.92e-11	E-GEOD-34773	purple
RTN2	negative	0.861	1.66e-08	E-GEOD-34773	darkgrey
RTN2	positive	-0.995	2.44e-24	E-GEOD-38012	turquoise
RYR1	positive	0.883	1.43e-04	E-GEOD-19102	sienna2
RYR1	positive	-0.994	2.21e-23	E-GEOD-38012	turquoise
SCN3B	positive	-0.879	1.67e-04	E-GEOD-19102	green3
SCN3B	positive	-0.956	5.85e-05	E-GEOD-38062	yellowgreen
SFT2D2	negative	-0.929	2.73e-16	E-GEOD-55272	black
SFT2D2	negative	0.975	8.14e-24	E-GEOD-55272	magenta
sgo-1	positive	-0.998	1.68e-13	E-GEOD-43864	blue
sgo-1	negative	0.985	3.18e-18	E-MTAB-1333	turquoise
SIK1B	negative	0.992	1.18e-06	E-GEOD-57739	turquoise
SIK1B	positive	0.989	1.13e-19	E-MTAB-1333	yellow
SIX4	negative	0.954	1.74e-12	E-GEOD-30505	red
SIX4	negative	-0.871	7.31e-09	E-GEOD-34773	greenyellow

SKP1	negative	0.995	2.19e-09	E-GEOD-32339	turquoise
SKP1	negative	0.940	9.01e-12	E-MTAB-1333	green
SKP1	negative	0.924	1.21e-10	E-MTAB-1333	green
SLC16A9	positive	0.989	1.02e-18	E-GEOD-30505	blue
SLC16A9	negative	-0.911	3.81e-05	E-MEXP-1810	darkmagenta
SLC7A11	negative	0.960	1.19e-13	E-MTAB-1333	blue
SLC7A11	positive	0.987	2.50e-09	EGEOD54853-CEL	brown
smz-2	positive	-0.970	3.19e-11	E-GEOD-42192	blue
smz-2	positive	0.988	2.18e-19	E-MTAB-1333	yellow
SNRPD1	negative	-0.875	1.91e-04	E-GEOD-19102	lightsteelblue1
SNRPD1	negative	0.954	6.08e-13	E-MTAB-1333	brown
somi-1	positive	0.917	2.74e-05	E-MEXP-1810	plum1
somi-1	positive	0.993	1.50e-10	EGEOD54853-CEL	yellow
spe-38	negative	-0.976	6.37e-08	E-GEOD-43864	darkorange
spe-38	negative	0.992	2.98e-10	E-GEOD-43864	lightgreen
SQSTM1	negative	0.959	1.07e-14	E-GEOD-34773	black
SQSTM1	negative	0.924	3.74e-04	E-GEOD-38062	yellowgreen
SQSTM1	positive	0.885	1.29e-04	E-MEXP-1810	lightcyan
sss-2	positive	-0.970	3.11e-11	E-GEOD-42192	blue
sss-2	positive	0.990	5.42e-20	E-MTAB-1333	yellow
T14G11.1	negative	-0.955	2.26e-04	E-GEOD-57739	darkorange
T14G11.1	negative	-0.909	7.81e-10	E-MTAB-1333	black
T19D12.2	negative	0.979	6.61e-04	E-GEOD-51502	turquoise
T19D12.2	positive	-0.892	9.42e-05	E-MEXP-1810	royalblue
T22B3.3	negative	0.998	3.30e-08	E-GEOD-57739	turquoise
T22B3.3	positive	0.989	1.42e-19	E-MTAB-1333	yellow
T28B11.1	positive	-0.928	1.39e-05	E-MEXP-1810	brown
T28B11.1	negative	0.964	3.65e-14	E-MTAB-1333	blue
T28H11.7	positive	-0.981	8.69e-13	E-GEOD-42192	blue
T28H11.7	positive	0.987	4.02e-19	E-MTAB-1333	yellow
TGIF2	negative	0.964	7.01e-06	E-GEOD-32339	pink
TGIF2	positive	0.883	1.08e-08	E-MTAB-1333	black
TMEM70	positive	-0.913	8.24e-11	E-GEOD-34773	darkgrey
TMEM70	negative	0.952	1.87e-06	E-MEXP-1810	brown
TNNI2	positive	0.905	5.22e-05	E-MEXP-1810	plum1
TNNI2	positive	0.870	2.34e-04	E-MEXP-1810	plum1
TNNI2	positive	0.992	3.43e-10	EGEOD54853-CEL	brown
TOMM70	positive	-0.917	2.70e-05	E-GEOD-19102	darkmagenta
TOMM70	positive	0.879	1.65e-04	E-GEOD-19102	deeppink
TUBG1	negative	0.948	1.03e-04	E-GEOD-38062	plum1
TUBG1	positive	0.987	1.43e-12	E-GEOD-66236	turquoise
UBE2E1	negative	-0.989	7.37e-19	E-GEOD-30505	blue
UBE2E1	negative	-0.993	8.90e-09	E-GEOD-32339	blue
UGT3A1	positive	-0.979	1.55e-12	E-GEOD-42192	turquoise
UGT3A1	positive	0.935	6.68e-04	E-GEOD-57739	sienna3
UTY	negative	0.986	2.97e-04	E-GEOD-51502	turquoise
UTY	positive	0.985	6.35e-09	EGEOD54853-CEL	yellow
VAMP3	negative	-0.912	9.32e-11	E-GEOD-34773	greenyellow
VAMP3	negative	-0.885	1.96e-09	E-GEOD-34773	tan
VRK2	positive	-0.972	1.70e-11	E-GEOD-42192	blue
VRK2	negative	-0.935	8.29e-06	E-GEOD-43864	darkorange
VRK2	negative	0.996	1.77e-07	E-GEOD-57739	turquoise
VRK2	negative	0.993	9.81e-07	E-GEOD-57739	turquoise
VRK2	negative	0.982	2.26e-17	E-MTAB-1333	turquoise

VRK2	positive	0.990	3.87e-20	E-MTAB-1333	yellow
VRK2	positive	0.989	1.44e-19	E-MTAB-1333	yellow
VRK2	positive	0.986	1.55e-18	E-MTAB-1333	yellow
W03G11.4	positive	0.993	1.43e-10	E-GEOD-43864	yellow
W03G11.4	positive	0.990	8.23e-10	E-GEOD-43864	yellow
W03G11.4	positive	0.998	3.84e-06	E-GEOD-9246	turquoise
W03G11.4	positive	0.990	6.47e-10	EGEOD54853-CEL	yellow
WDR47	negative	-0.993	6.36e-05	E-GEOD-51502	black
WDR47	positive	0.947	2.94e-06	E-MEXP-1810	darkmagenta
WIPF1	negative	0.954	1.55e-06	E-GEOD-19102	blue
WIPF1	negative	0.919	3.60e-11	E-GEOD-34773	magenta
WIPF1	negative	0.962	6.73e-14	E-MTAB-1333	blue
Y42H9AR.4	negative	0.942	2.15e-11	E-GEOD-30505	black
Y42H9AR.4	negative	-0.996	1.78e-09	E-GEOD-32339	blue
ZC3H15	negative	0.982	1.56e-08	E-GEOD-43864	midnightblue
ZC3H15	negative	-0.978	7.07e-25	E-GEOD-55272	yellow
ZFP36	negative	-0.994	4.29e-09	E-GEOD-32339	blue
ZFP36	negative	-0.994	4.60e-09	E-GEOD-32339	blue
ZFP36	positive	0.971	1.33e-05	E-GEOD-8696	red

Supplement Table 3: Hub genes found by quantile normalization with an export of 60 genes per health-associated module. *67 genes were identified as hub genes with the same WGCNA cross-species consensus protocol when a quantile normalization was performed on the expression data. Only 13 genes were found in a cross-species consensus when 30 genes were exported.*

ABCD4	worm,human
ABCE1	mouse,worm
AKR7L	mouse,rat
AL669918.1	mouse,worm
ANXA2	mouse,human
ATF2	mouse,worm
BAP1	mouse,worm
BRD8	mouse,worm
C4BPA	mouse,rat
CAPN7	worm,mouse
CASP1	mouse,rat
CEBPB	worm,mouse
DAB2	mouse,worm
DIMT1	mouse,worm
EIF5A	mouse,worm
ELOA	mouse,rat
EXOC6	mouse,worm
FBXO32	mouse,human
FKBP9	mouse,rat
GBP2	mouse,rat
GDF15	mouse,human
GPSM3	mouse,rat
GRASP	mouse,worm
HIST1H2BA	mouse,worm
HSPB3	mouse,human
IDH3A	rat,worm
ILF2	mouse,human
ITM2A	worm,mouse
LUZP1	rat,worm
MAP2K5	worm,human

MED17	worm,mouse
MFN1	mouse,rat
MRPL12	mouse,worm
MRPL51	mouse,worm
NAA25	mouse,worm
NFYA	worm,mouse
NKG7	mouse,rat
OGA	rat,human
OR2Y1	mouse,rat
PDIA4	mouse,rat
PIPOX	mouse,worm
PLD4	worm,mouse
PPP1CA	worm,human
PPP3CB	mouse,worm
PRKCZ	mouse,worm
PRPF38A	mouse,worm
PSMB9	mouse,rat
PSMD12	mouse,rat
RAB8B	mouse,worm
RAMP1	human,rat
RMI1	mouse,worm
RMND5A	worm,mouse
RPL13A	mouse,worm
RTN2	mouse,human
SCLY	mouse,rat
SCN1B	human,rat
SDCBP	mouse,worm
SDF2	mouse,worm
SERPINF1	mouse,rat
SKP1	rat,worm
SQOR	worm,mouse
SQSTM1	mouse,worm
SRD5A2	mouse,worm
SSRP1	mouse,worm
TRUB2	mouse,worm
UBC	mouse,worm
UBE4B	worm,mouse

Supplement Table 4: Genes in interactions found by quantile normalization with an export of 60 genes per health-associated module. *44 genes were identified as hub genes with the same WGCNA cross-species consensus protocol when a quantile normalization was performed on the expression data. Only 10 genes were found in a cross-species consensus when 30 genes were exported per module. This was not found to be significant in a permutation test.*

AASDHPPT	mouse,worm
ABCE1	mouse,worm
ACOT9	mouse,worm
ACVR1	worm,mouse
AL669918.1	mouse,worm
ANTKMT	worm,mouse
BAP1	mouse,worm
BCS1L	rat,worm
CAMK2B	human,rat
CEBPB	worm,mouse
CETN2	worm,rat
CUL1	mouse,worm

DAB2	mouse,worm
DAG1	mouse,worm
DBH	mouse,worm
EDF1	mouse,worm
FAAH	mouse,worm
FRA10AC1	mouse,worm
GTPBP10	mouse,worm
ITGA3	worm,mouse
KCNK6	mouse,worm
LMBR1L	worm,mouse
LONRF1	mouse,worm
MED11	mouse,worm
MINDY2	worm,mouse
MRPL19	mouse,worm
NF2	worm,mouse
OXSM	mouse,worm
PAXIP1	mouse,worm
PFDN1	worm,mouse
PPP1CA	worm,human
PRPF38A	mouse,worm
RFK	worm,mouse
RMND5A	mouse,worm
RNF7	worm,mouse
SLC35B1	worm,human
SLC38A10	worm,mouse
SOX2	mouse,worm
SSRP1	mouse,worm
STAT1	mouse,worm
TIMP3	mouse,worm
VPS11	mouse,worm
ILF2	mouse,human,worm
